# Genes and Pathways Implicated in Tetralogy of Fallot Revealed by Ultra-Rare Variant Burden Analysis in 231 Genome Sequences

**DOI:** 10.3389/fgene.2020.00957

**Published:** 2020-09-15

**Authors:** Roozbeh Manshaei, Daniele Merico, Miriam S. Reuter, Worrawat Engchuan, Bahareh A. Mojarad, Rajiv Chaturvedi, Tracy Heung, Giovanna Pellecchia, Mehdi Zarrei, Thomas Nalpathamkalam, Reem Khan, John B. A. Okello, Eriskay Liston, Meredith Curtis, Ryan K. C. Yuen, Christian R. Marshall, Rebekah K. Jobling, Erwin Oechslin, Rachel M. Wald, Candice K. Silversides, Stephen W. Scherer, Raymond H. Kim, Anne S. Bassett

**Affiliations:** ^1^Ted Rogers Centre for Heart Research, Cardiac Genome Clinic, The Hospital for Sick Children, Toronto, ON, Canada; ^2^Deep Genomics Inc., Toronto, ON, Canada; ^3^The Centre for Applied Genomics, The Hospital for Sick Children, Toronto, ON, Canada; ^4^Program in Genetics and Genome Biology, The Hospital for Sick Children, Toronto, ON, Canada; ^5^Labatt Heart Centre, Division of Cardiology, The Hospital for Sick Children, Toronto, ON, Canada; ^6^Clinical Genetics Research Program, Centre for Addiction and Mental Health, Toronto, ON, Canada; ^7^The Dalglish Family 22q Clinic, University Health Network, Toronto, ON, Canada; ^8^Department of Molecular Genetics, University of Toronto, Toronto, ON, Canada; ^9^Genome Diagnostics, Department of Pediatric Laboratory Medicine, The Hospital for Sick Children, Toronto, ON, Canada; ^10^Centre for Genetic Medicine, The Hospital for Sick Children, Toronto, ON, Canada; ^11^Laboratory Medicine and Pathobiology, University of Toronto, Toronto, ON, Canada; ^12^Division of Cardiology, Toronto Congenital Cardiac Centre for Adults at the Peter Munk Cardiac Centre, Department of Medicine, University Health Network, Toronto, ON, Canada; ^13^Division of Clinical and Metabolic Genetics, The Hospital for Sick Children, Toronto, ON, Canada; ^14^Fred A. Litwin Family Centre in Genetic Medicine, University Health Network, Department of Medicine, University of Toronto, Toronto, ON, Canada; ^15^Department of Psychiatry, University of Toronto, Toronto, ON, Canada; ^16^Department of Mental Health, Toronto General Hospital Research Institute, University Health Network, Toronto, ON, Canada

**Keywords:** tetralogy of fallot, heart disease, whole genome sequencing, NOTCH1, FLT4, rare variants

## Abstract

Recent genome-wide studies of rare genetic variants have begun to implicate novel mechanisms for tetralogy of Fallot (TOF), a severe congenital heart defect (CHD). To provide statistical support for case-only data without parental genomes, we re-analyzed genome sequences of 231 individuals with TOF (*n* = 175) or related CHD. We adapted a burden test originally developed for *de novo* variants to assess ultra-rare variant burden in individual genes, and in gene-sets corresponding to functional pathways and mouse phenotypes, accounting for highly correlated gene-sets and for multiple testing. For truncating variants, the gene burden test confirmed significant burden in *FLT4* (Bonferroni corrected *p*-value < 0.01). For missense variants, burden in *NOTCH1* achieved genome-wide significance only when restricted to constrained genes (i.e., under negative selection, Bonferroni corrected *p*-value = 0.004), and showed enrichment for variants affecting the extracellular domain, especially those disrupting cysteine residues forming disulfide bonds (OR = 39.8 vs. gnomAD). Individuals with *NOTCH1* ultra-rare missense variants, all with TOF, were enriched for positive family history of CHD. Other genes not previously implicated in CHD had more modest statistical support in gene burden tests. Gene-set burden tests for truncating variants identified a cluster of pathways corresponding to VEGF signaling (*FDR* = 0%), and of mouse phenotypes corresponding to abnormal vasculature (*FDR* = 0.8%); these suggested additional candidate genes not previously identified (e.g., *WNT5A* and *ZFAND5*). Results for the most promising genes were driven by the TOF subset of the cohort. The findings support the importance of ultra-rare variants disrupting genes involved in VEGF and NOTCH signaling in the genetic architecture of TOF, accounting for 11–14% of individuals in the TOF cohort. These proof-of-principle data indicate that this statistical methodology could assist in analyzing case-only sequencing data in which ultra-rare variants, whether *de novo* or inherited, contribute to the genetic etiopathogenesis of a complex disorder.

## Introduction

Congenital heart defects (CHD) occur in 8/1000 live births and are a leading cause of mortality from birth defects (Glidewell et al., 2019), with a wide spectrum of severity (Zaidi and Brueckner, 2017). Among CHD, tetralogy of Fallot (TOF) is the most common of the more severe (cyanotic) conditions. Individuals with TOF present with a combination of abnormalities (pulmonary valve stenosis, right ventricular hypertrophy, ventricular septal defect and overriding aorta) that together lead to insufficient tissue oxygenation. Genetic factors are major contributors to the etiology of TOF; 20% of patients have pathogenic copy number variants (CNV) or larger chromosomal anomalies (Mercer-Rosa et al., 2015; Morgenthau and Frishman, 2018). Recent studies have also begun to elucidate the genome-wide role of rare variants at the sequence level, including substitutions and small insertions/deletions.

In a multi-center exome sequencing study of various CHD that focused on loss-of-function variants and included parental sequencing data enabling *de novo* variant identification, the TOF sub-group drove a significant genome-wide burden finding (*p*-value ≤ 1.3 × 10^–6^) of *de novo* and ultra-rare inherited (allele frequency ≤ 1 × 10^–5^) heterozygous truncating variants for a novel gene, *FLT4* (Jin et al., 2017). Of the nine probands with *FLT4* truncating variants, corresponding to 2.3% of the TOF group, 7 were inherited with evidence of incomplete penetrance (Jin et al., 2017).

In an independent case-only study, but using whole genome sequencing (WGS) (Reuter et al., 2019), we investigated 175 adults with TOF for ultra-rare loss-of-function variants (including structural variants) disrupting *FLT4* and other vascular endothelial growth factor (VEGF) pathway genes predicted to be haploinsufficient based on the ExAC pLI index (Lek et al., 2016). We identified seven truncating variants in *FLT4*, two in *KDR*, and one each in *BCAR1*, *FGD5*, *FOXO1*, *IQGAP1* and *PRDM1*, corresponding in aggregate to 8.0% of participants (Reuter et al., 2019); all variants were absent from public databases. The results suggested the importance of VEGF signaling; however, the statistical burden of ultra-rare variants was not systematically investigated (Reuter et al., 2019).

Another recent multi-center exome sequencing study of 829 patients with TOF reported genome-wide significant (*p*-value ≤ 5 × 10^–8^) excess of ultra-rare (absent from a public exome database and other reference data) deleterious variants for *FLT4* and *NOTCH1* (Page et al., 2019). Loss-of-function variants predominated for *FLT4*, and missense variants for *NOTCH1* (Page et al., 2019).

In the current study, we undertook a comprehensive statistical re-analysis of the cohort with WGS data that we had previously investigated by manual curation for ultra-rare truncating variants in TOF, reporting those in the VEGF pathway (Reuter et al., 2019). In an attempt to boost power, we included the sequencing data available for 56 CHD cases as well as for the original 175 TOF cases (*n* = 231 total). Following the precedent set by previous studies, we focused on ultra-rare truncating (stop-gain, frameshift and splice site altering) and ultra-rare missense variants that were not reported in the gnomAD database (Karczewski et al., 2019), and were identified in only one proband, i.e., singletons in this CHD cohort. We tested burden by adapting a test originally developed for *de novo* variants by rescaling the mutation probability for ultra-rare variants. Since ultra-rare variants are enriched in *de novo* variants and those likely to have arisen recently, this is an appropriate extension of the test. To boost power, we additionally tested gene-sets corresponding to (a) functional pathways, derived from Gene Ontology (GO) (Carlson, 2019), BioCarta^1^, Kyoto Encyclopedia of Genes and Genomes (KEGG) (Kanehisa et al., 2017)^2^, REACTOME (Fabregat et al., 2018), NCI-Nature Pathway Interaction Database (PID)^3^; and (b) phenotypes in mouse orthologs, derived from Mouse Genome Informatics (MGI) and based on the Mouse Phenotype Ontology (MPO) classification (Bult et al., 2019). To control for correlations between highly overlapping gene-sets that could lead to incorrect multiple *p*-value corrections, we adopted a greedy step-down approach to cluster gene-sets with highly overlapping genes. A sampling-based false discovery rate (FDR) was then estimated. We did not analyze structural variants because no broadly accepted probabilistic framework has yet been developed to determine the statistical significance of their burden.

## Results

### Identification of Ultra-Rare Variants

Variant calls from the CHD WGS data-set were filtered to retain only high-quality ultra-rare variants that were found in only one of the 231 CHD adult probands studied, but not in gnomAD; these were then categorized as truncating or missense based on their effect on the principal transcript (see section “Materials and Methods” for details). With respect to the 2,003 truncating ultra-rare variants initially identified, 868 variants remained after applying the low quality and frameshift indel filter, 764 after applying the principal transcript effect filter, 752 after applying the splice site alteration filter, and finally 642 after considering a maximum of one ultra-rare variant per gene per subject. For the 4,324 missense variants initially identified, 3,521 remained after applying the low-quality filter, 3,359 after applying the principal transcript filter, and finally, 3,293 ultra-rare missense variants after considering a maximum of one ultra-rare variant per gene per subject. These variants will be referred to as ultra-rare variants.

**FIGURE 1:**
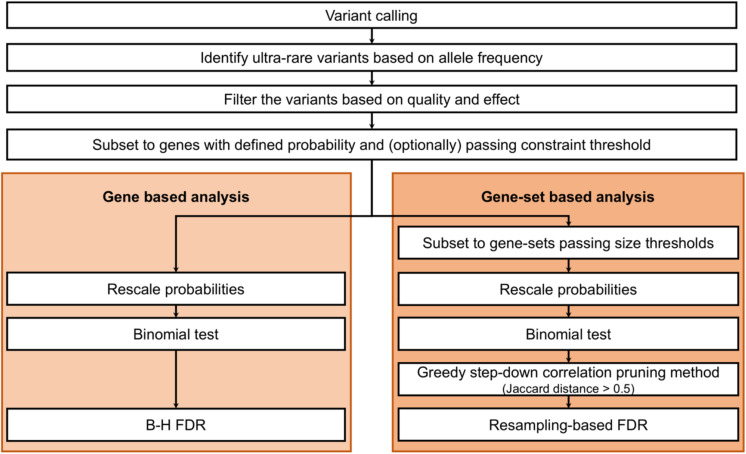
General gene and gene-set burden analyses overview.

We tested these ultra-rare truncating and missense variants for gene and gene-set burden (see Figure 1 for an overview of the analysis workflow; all ultra-rare variants identified are listed in Supplementary Table S1). For all analyses, we tested truncating and missense variants separately because of the likely differences in the genetic architecture of these variant types. Bonferroni multiple test correction was then performed jointly for both variant types (binomial burden test results) and FDR multiple test corrections were performed separately for each variant type.

### Gene Burden Results

Genes were tested separately for the burden of ultra-rare truncating and missense variants, using a binomial test based on rescaled *de novo* mutation probabilities (as described in the section “Materials and Methods”). We performed multiple test correction on all genes with a defined probability, and also on a more constrained subset: for truncating variants, gnomAD pLoF o/e < 0.35; for missense variants, gnomAD missense o/e < 0.75 (where pLoF indicates predicted to result in complete protein loss of function, and *o/e* indicates observed/expected; see Supplementary Figure S1 for the relation between *pLI* and missense *z-score* constraint indices). Constrained genes are presumed to more likely contribute to disease, since they are under negative selection. Here, the thresholds were specifically set to include moderately constrained genes, considering the incomplete penetrance observed for TOF (Jin et al., 2017; Page et al., 2019; Reuter et al., 2019). There were 603 genes with at least one ultra-rare truncating variant, of which 163 passed the constraint threshold; there were 2801 genes with at least one ultra-rare missense variant, of which 739 passed the constraint threshold (see Supplementary Tables S2, S3, respectively, for details). To assess the validity of the gene burden results, we performed several additional analyses: (a) we checked the distribution of observed versus expected *p*-values, to monitor for systematic *p*-value inflation; (b) we compared the *p*-values obtained for CHD to those obtained for WGS data available for 263 individuals with schizophrenia, processed in exactly the same way; and (c) we reassessed the gene burden by comparing to gnomAD singletons (i.e., variants in genes with only one allele count in the gnomAD data-set).

**FIGURE 2:**
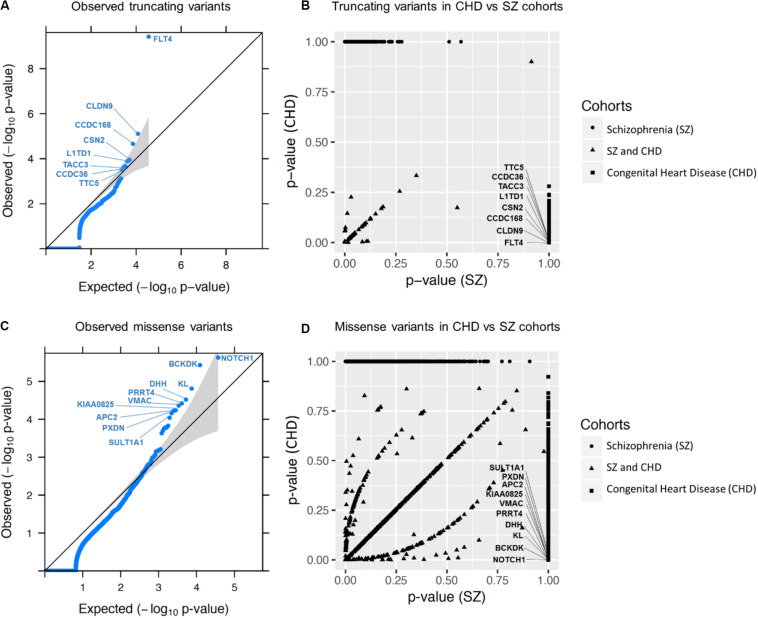
Gene burden analysis results for all genes. **(A,C)** show the quantile-quantile (QQ) plots obtained for all ultra-rare truncating and missense variants in CHD, respectively (i.e., not setting any gene constraint cutoff). The QQ plots represent the scatter plots of the –log10 (*p*-value) expected under the null hypothesis of no genetic association versus the observed –log10 (*p*-value) for all 231 CHD samples. Gray shading indicates the 95% confidence interval. **(B,D)** represent scatter plots of gene burden *p*-values for truncating and missense variants, respectively, comparing the CHD and schizophrenia WGS data. Names of the top 8 and 10 genes identified for truncating **(A)** and missense **(C)** variants, respectively, are shown (results for top 6 of each are presented in Table 1); FLT4 **(A)** and NOTCH1 **(C)** were the most significant genes identified, neither with any observation in the comparison schizophrenia cohort **(B,D)**. These plots were generated based on the genes without constraint on o/e score. Supplementary Figure S4 shows results for genes with constraint.

For truncating variants, there was only one constrained gene (of the 163 with at least one ultra-rare truncating variant) with significant variant burden: *FLT4* (uncorrected *p*-value = 9.56 × 10^–12^, BH-FDR = 6.99 × 10^–8^, Bonferroni-corrected *p*-value = 1.19 × 10^–7^). When testing all genes (i.e., the 603 genes with at least one ultra-rare truncating variant), in addition to *FLT4*, we identified *CLDN9* as significantly enriched with ultra-rare variants at the FDR threshold of 10% (uncorrected *p*-value = 7.80 × 10^–6^, BH-FDR = 0.072, Bonferroni-corrected *p*-value = 0.293) (see Table 1 and Supplementary Table S2 for all details). There was no evidence of genome-wide inflation in either the all genes or constrained genes subset analysis (see Figure 2 and Supplementary Figure S2).

**TABLE 1:** Top significant genes (BH-FDR < 10%) with ultra-rare variants identified in 231 individuals with CHD, as inferred from gene-based burden analyses for truncating and missense ultra-rare variants, respectively, with and without using a gene constraint cut-off.

Gene Name	Number of observed variants^1^	All genes, no constraint		Genes with constraint^2^	
		*P*-value	BH-FDR^3^	*P*-value	BH-FDR^3^
**Truncating variants**					
FLT4	7	3.84×10^−10^	7.15×10^−6^	9.56×10^−12^	6.99×10^−8^
*CLDN9*	2	7.80×10^−6^	0.0726	NA	NA

**Missense variants**					
NOTCH1	8	8.88×10^−7^	0.0168	3.52×10^−7^	0.0018
*BCKDK*	4	2.21×10^−6^	0.0210	1.35×10^−6^	0.0035
*KL*	4	9.25×10^−5^	0.0585	NA	NA
*DHH*	3	2.05×10^−5^	0.0882	1.42×10^−5^	0.0245
*PRRT4*	3	2.57×10^−5^	0.0882	NA	NA
*VMAC*	2	3.34×10^−5^	0.0882	NA	NA
*KIAA0825*	3	3.95×10^−5^	0.0882	NA	NA
*APC2*	4	3.65×10^−5^	0.0882	NA	NA
*PXDN*	4	4.19×10^−5^	0.0882	NA	NA

*^1^All observed variants were in individuals with TOF, except 1 each in genes *KL*, *DHH*, *PRRT4, KIAA0825*, and *APC2*, for missense variants. ^2^Only those variants in genes deemed to be constrained, i.e., in genes with o/e score in gnomAD < 0.35 for truncating variants and <0.75 for missense variants, are considered. Note that not all protein-coding genes have these data available. ^3^The *Benjamini-Hochberg* False Discovery Rate. NA = not available, indicating that the respective gene was not in the gene list, thus data were not available. Selected Bonferroni-corrected (genome-wide) p-values are provided in the text.*

Considering the top-associated CHD genes without using a constraint threshold, none had a similar *p*-value in the schizophrenia sequencing data. When applying the constraint threshold, a single top-associated gene that failed the 10% BH-FDR threshold (*ATXN3*) appeared to have a somewhat similar *p*-value for schizophrenia. However, visualization of the bam files re-classified those variants in both CHD and schizophrenia cohorts to be in-frame polymorphisms (see Supplementary Table S4). For *FLT4* and *CLDN9*, where BH-FDR was under the 10% threshold, we evaluated the truncating ultra-rare variant burden in CHD compared to that in gnomAD: *FLT4* had an even more significant association (uncorrected *p*-value = 2.43 × 10^–15^, BH-FDR = 4.01 × 10^–11^), whereas *CLDN9* was less significant (uncorrected *p*-value = 7.8 × 10^–4^, BH-FDR = 1), leading us to question the validity of CDLN9’s association to CHD (see Supplementary Table S5 and Supplementary Figure S3). Restricting to constrained genes may have some utility in prioritizing genes, but these results are too limited to draw robust general conclusions.

Of the 739 genes with ultra-rare missense variants that passed the constraint threshold, the following 3 passed the 10% FDR threshold: *NOTCH1* (uncorrected *p*-value = 3.52 × 10^–7^, BH-FDR = 0.0018, Bonferroni-corrected *p*-value = 0.0044), *BCKDK* (uncorrected *p*-value = 1.35 × 10^–6^, BH-FDR = 0.0035, Bonferroni-corrected *p*-value = 0.0169), *DHH* (uncorrected *p*-value = 1.42 × 10^–5^, BH-FDR = 0.0245, Bonferroni-corrected *p*-value = 0.177); see Table 1 and Supplementary Table S3 for further details. When considering all 2,801 genes with ultra-rare missense variants, regardless of constraint, the BH-FDR for gene *DHH* (0.088) was less significant, but other genes, *KL*, *PRRT4*, *VMAC*, *KIAA0825*, *APC2*, and *PXDN*, passed the 10% BH-FDR cut-off (*KL* BH-FDR = 0.058, other genes BH-FDR = 0.088) (see Table 1 and Supplementary Table S3). There was no evidence of genome-wide inflation in either analysis (see Figure 2 and Supplementary Table S4).

When applying the constraint threshold, there was one top-associated gene for CHD that did not meet the 10% BH-FDR threshold and that had somewhat similar results in the schizophrenia cohort (*OLIG2*: CHD uncorrected *p*-value = 1.39 × 10^–4^, BH-FDR = 0.181; schizophrenia uncorrected *p*-value = 0.017) (see Supplementary Table S6). *OLIG2* was also less significant in the gnomAD singleton variant comparison (*p*-value = 5.22 × 10^–3^), thus indicating its questionable validity for CHD. For the genes identified without using the constraint threshold, none had a similar *p*-value for missense variants in schizophrenia. Comparing the missense ultra-rare variant burden in CHD to the singleton burden in gnomAD, only *NOTCH1* passed the BH-FDR 10% threshold, with a similar uncorrected *p*-value (see Supplementary Table S5 and Supplementary Figure S4). The main benefit of restricting the analysis to missense-constrained genes appeared to be an increased significance for *NOTCH1* after multiple test correction.

### Gene-Set Burden Results – Truncating Variants

Restricting to genes constrained for truncating variants, the gene-set burden analysis (as described in the section “Materials and Methods”) identified one cluster for GO/pathways, and one cluster for MPO, both of which were significant at the sampling FDR < 10%. The FDR approached 1.0 (non-significant) for other clusters (see Table 2). Gene-set sub-clusters were manually identified with the aid of the Cytoscape app EnrichmentMap (Reimand et al., 2019) (see Table 3 and Supplementary Figure S5). The GO/pathways cluster (uncorrected *p*-value = 5.39 × 10^–13^, sampling-based FDR = 0) comprised 30 gene-sets, 20 of which were clearly related to VEGF signaling and/or blood vessel development (angiogenesis). *FLT4* was by far the most significant gene (truncating variants *n* = 7, uncorrected *p*-value = 9.56 × 10^–12^), with other genes such as *KDR* (truncating variants *n* = 2, uncorrected *p*-value = 0.001), *FOXO1* (truncating variant *n* = 1, uncorrected *p*-value = 0.008), *ZFAND5* (truncating variant *n* = 1, uncorrected *p*-value = 0.008) and *WNT5A* (truncating variant *n* = 1, uncorrected *p*-value = 0.010) having more modest contributions (see Table 3 and Supplementary Tables S7, S8). The MPO cluster (uncorrected *p*-value = 9.64 × 10^–11^, sampling-based FDR = 0.008) comprised 19 gene-sets, 15 of which corresponded to abnormalities of the cardiovascular system such as abnormal vessel morphology and cardiac-related bleeding in mice (see Table 3 and Supplementary Tables S9, S10). The GO pathway and MPO cluster results additionally identified other potential candidate genes for TOF associated with functions of *FLT4* that were not identified in the previous study, including *AKAP12*, *PKD1*, *ATF2*, and *EPN1* (Table 3). While other clusters were not significant after multiple test correction, some top-scoring clusters had a clear functional or phenotypic relation to CHD (for instance, planar cell polarity in neural tube closure, ranking third for GO and GO/pathways; positive regulation of vascular smooth cell migration, ranking fourth for GO/pathways) and including additional promising candidate genes (e.g., *DVL3*, *KIF3A*) (see Supplementary Table S7).

**TABLE 2:** Top six gene-set clusters for truncating ultra-rare variant burden analyses in CHD using Gene Ontology (GO)/pathways and Mouse Phenotype Ontology (MPO), and restricting to constrained genes.

Gene-set clusters	Observed truncating variants in constrained genes (o/e score in gnomAD < 0.35)	*P*-value	Resampling based FDR
**GO/pathways**			
VEGF signaling and blood vessel development	8	5.39×10^−13^	0
Ion antiporter activity	5	0.0005	0.9564
Planar cell polarity pathway involved in neural tube closure	3	0.0013	0.9564
Positive regulation of vascular associated smooth muscle cell migration	4	0.0017	0.9564
Peptidyl-tyrosine autophorphorylation	5	0.0027	0.9564
Protein quality control for misfolded or incompletely synthesized proteins	3	0.0029	0.9564

**MPO**			
Abnormal lymphangiogenesis	7	9.64×10^−11^	0.0080
Abnormal cranial neural crest cell morphology	3	0.0010	0.9605
Neuronal cytoplasmic inclusions	2	0.0022	0.9605
Absent pharyngeal arches	4	0.0031	0.9605
Abnormal CD5-positive T cell number	2	0.0036	0.9605
Cochlear ganglion degeneration	4	0.0037	0.9605

**TABLE 3:** Gene-set sub-clusters derived from the two gene-set clusters with significant truncating ultra-rare burden from Table 2.

Most significant composite gene-set sub-clusters	*P*-value	Genes^1^ (contributing number of variants, *p*-value)
**GO/pathways**		
Positive regulation of protein kinase C signaling	5.39×10^−13^	*FLT4*^2^, *WNT5A* (1, 0.010)
Regulation of protein kinase C signaling	1.17×10^−12^	*FLT4*^2^, *WNT5A* (1, 0.010), *AKAP12* (1, 0.024)
VEGF and related pathways, and transmembrane receptor protein kinase activity	1.68×10^−12^	*FLT4*^2^, *KDR*^3^ (2, 0.001)
Regulation of blood vessel remodeling, VEGFR3 signaling in lymphatic endothelium, and lung alveolus development	4.70×10^−10^	*FLT4*^2^
Lymph vessel morphogenesis and development	6.73×10^−9^	*FLT4*^2^, *PKD1* (1, 0.046)
Respiratory system process and gaseous exchange	4.73×10^−8^	*FLT4*^2^, *ZFAND5* (1, 0.008)
Endothelial cell proliferation and migration	6.45×10^−7^	*FLT4*^2^, *KDR*^3^ (2, 0.001), *WNT5A* (1, 0.010)

**MPO**		
Anterior cardinal vein development, abnormal lymph circulation, abnormal lymphatic system physiology, and ascites	9.64×10^−11^	*FLT4*^2^
Abnormal lymphangiogenesis and abnormal lymphatic vessel morphology	1.55×10^−10^	*FLT4*^2^, *KDR*^3^ (2, 0.001)
Heart hemorrhage	4.20×10^−7^	*FLT4*^2^, *KDR*^3^ (2, 0.001), *ATF2* (1, 0.036), *PKD1* (1, 0.046)
Hemopericardium	2.33×10^−6^	*FLT4*^2^, *ATF2* (1, 0.036), *PKD1* (1, 0.046)
Skin edema and hydrops fetalis	5.84×10^−5^	*FLT4*^2^, *PKD1* (1, 0.046)
Abnormal vitelline vascular remodeling	2.41×10^−4^	*FLT4*^2^, *KDR*^3^ (2, 0.001), *FOXO1*^3^ (1, 0.008), *EPN1* (1, 0.015), *TTN* (1, 0.740)

*^1^Genes listed are all those meeting the constraint threshold of o/e < 0.35, e.g., including genes not reaching significance (e.g., *TTN*). ^2^For each significant gene-set subcluster, for gene *FLT4* the number of truncating variants contributing is 7, and the *p*-value is 9.56×10^−12^. ^3^Candidate genes previously identified through manual curation methods, relevant to the VEGF pathway, in addition to *FLT4* (Reuter et al., 2019).*

Since *FLT4* had such a prominent role in driving the gene-set signal for truncating variants, we repeated the analysis without *FLT4*. No significant gene-set cluster was identified.

Similar results were obtained when considering all genes (i.e., without restricting to constrained genes), but the MPO cluster had FDR just slightly higher than the 10% threshold (see Supplementary Table S9).

For the missense variant analysis, we observed no significant gene-sets, with or without applying the constraint cut-off (see Supplementary Tables S11, S12).

### Detailed *in silico* Analysis of Ultra-Rare Missense Variants in *NOTCH1* and Other Genes

Given that our previous report had focused on ultra-rare truncating variants (Reuter et al., 2019), we reviewed in detail the ultra-rare missense variants identified, considering amino acid conservation in orthologous vertebrate sequences and *in silico* predictors (SIFT, PolyPhen2, and Mutation Assessor) (Adzhubei et al., 2010; Reva et al., 2011; Vaser et al., 2016). For *NOTCH1*, this manual review deemed seven of the eight ultra-rare missense variants to be either likely deleterious (*n* = 6) or potentially deleterious (*n* = 1). For *BCKDK*, one of four was likely deleterious, and one of four potentially deleterious; for *KL*, three of four were potentially deleterious; for *DHH*, one of three was likely deleterious and one of three potentially deleterious; see Supplementary Table S13).

**FIGURE 3:**
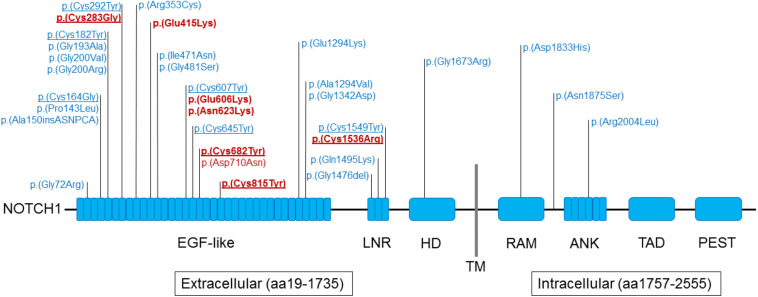
Schematic representation of NOTCH1 domains and rare variants identified in individuals with tetralogy of Fallot. Findings from the current study involving 8 of 175 probands with TOF are indicated in red font; 24 ultra-rare missense variants from the Page et al. study (Page et al., 2019) are indicated in blue font. The seven ultra-rare missense NOTCH1 variants deemed to be either likely deleterious (*n* = 6) or potentially deleterious (p.Asn623Lys) are indicated in bold red font (details in Supplementary Table S13). Underline indicates those variants that alter evolutionarily conserved cysteine residues; eight located within the EGF-like repeats domain and two in the LNR (Lin12-Notch) domain. Abbreviations: aa, amino acid; ANK, ankyrin; EGF, epidermal growth factor; HD, heterodimerization domain; LNR, Lin/NOTCH repeats; PEST, sequence rich in proline, glutamic acid, serine, and threonine; RAM, RBP-JK–associated molecule region; TAD, transactivation domain; TM, transmembrane domain (aa1736–1756). The protein domains were derived from UniProt (https://www.uniprot.org/uniprot/P46531).

All 8 *NOTCH1* variants identified reside in the extracellular domain of the encoded protein (amino acids 19-1735, see Figure 3), compared to 958 of 1,413 gnomAD v2.1 ultra-rare missense variants (one-sided Fisher’s Exact Test *p*-value = 0.045, odds ratio = + Inf). Similar to previously reported exome sequencing findings (Page et al., 2019), four of these eight variants alter evolutionarily conserved cysteine residues that establish disulfide bonds, located within the EGF-like repeats or the LNR (Lin12-Notch) domain (Wouters et al., 2005; Gordon et al., 2009). This represents highly significant enrichment compared to such variants from gnomAD v2.1 (23 of 958 variants; one-sided Fisher’s Exact Test *p*-value = 3.15 × 10^–5^, odds ratio = 39.8).

Notably, all 8 of the ultra-rare missense variants in *NOTCH1* were identified within the 175 individuals with TOF, representing 4.6% of those studied. There was significant enrichment for positive family history of CHD compared to the rest of the TOF sample (four of eight probands; two-sided Fisher’s Exact Test *p*-value = 0.003431, odds ratio = 11.49). Details of phenotype and family history are provided for individuals with these 8 *NOTCH1* and 12 other (truncating) variants in Supplementary Table S14. None of these adults with *NOTCH1* ultra-rare variants had a history of a clinical diagnosis of Adams-Oliver syndrome (AOS5, OMIM: 616028), a rare multi-system developmental syndrome associated with pathogenic *NOTCH1* variants and mechanism proposed to involve vascular disruption.

### Focus on the TOF Subset

Ultra-rare variants in the genes that were highly significant for gene burden (*FLT4*, *NOTCH1*) or that were suggested by the gene-set analysis (*FOXO1*, *KDR*, *WNT5A*, *ZFAND5*) all occurred in individuals with TOF (see Supplementary Table S14). For this reason, we repeated the gene burden analyses for the TOF subset (*n* = 175). *FLT4* and *NOTCH1* gene burden results were more significant, whereas KL and DHH were less significant; no additional gene passed the Bonferroni correction (see Supplementary Table S15). We also repeated the gene-set burden tests for the TOF subset. Truncating ultra-rare variants produced very similar results. For ultra-rare missense variants, a large cluster of MPO gene-sets was significant (FDR = 0.056) and two GO/pathways clusters were borderline significant (FDR < 25%). The significance of all of these clusters was driven by *NOTCH1*, and further examination revealed that they contained multiple cardiovascular-related gene-sets. Potentially interesting candidate genes with ultra-rare missense variants identified in these gene-set clusters included *ACVR2B*, *BMPR2*, *EGR3*, *ERG*, *FZD7*, *HDAC5*, *MEIS1*, *MIB1*, *MYH10*, *PRKCA*, *ROCK2*, *S1PR1*, *VASH1* and others with less evidence, further strengthening a connection of TOF to plausible mechanisms (e.g., VEGF, angiogenesis-related; see Supplementary Table S16).

### Results for Power Analyses

We performed power calculations to determine what sample size would be required to pass a Bonferroni-corrected *p*-value of 0.05 for genes with the same expected mutation probability and the same observed number of ultra-rare variants as *KDR*, *FOXO1*, *WNT5A* and *ZFAND5* for truncating variants and the same as *BCKDK*, *DHH* and *KL* for missense variants. For *KDR*, >80% power was achieved for ∼ 600 TOF subjects. For *FOXO1*, *WNT5A* and *ZFAND5*, >80% power was achieved for ∼ 1,600 TOF subjects (see Supplementary Figure S6A). For *BCKDK*, *DHH* and *KL* missense variants, >80% power was achieved for ∼ 600–750 CHD subjects (as shown in Supplementary Figure S6B).

## Discussion

In this study, we re-analyzed WGS data available for 231 individuals with CHD, including 175 with TOF, to extend previously published results (Reuter et al., 2019) by including ultra-rare missense variants and by using a statistical method modified to suit such case-only data. By rescaling *de novo* mutation probabilities for ultra-rare variants, we adapted a burden test originally developed for *de novo* variants, and tested truncating and missense ultra-rare variants separately for increased burden in genes and in functionally relevant gene-sets, using case-only data.

Previous results suggested that ultra-rare non-synonymous variants make an important contribution to the genetic etiology of CHD, especially to TOF (Jin et al., 2017; Page et al., 2019; Reuter et al., 2019). Since constrained genes (those known to be under negative selection) may be more likely to contribute to disease, in order to maximize power, we performed multiple test correction for all genes, and (separately) only for genes passing a constraint threshold tailored to the variant type and allowing for expected reduced penetrance in CHD. We assessed the validity of our results by ensuring the absence of inflation when considering the burden test *p*-value distribution. In addition, we compared burden results for CHD to a schizophrenia WGS data-set processed in the same way (including variant calling and QC), to help identify potential artifacts. Finally, we also retested burden by comparing results to gnomAD singletons that had been processed in the same way with respect to variant annotation.

### Gene Burden

For truncating ultra-rare variant burden, *FLT4* passed a stringent significance threshold of 0.01 after Bonferroni correction. For ultra-rare missense variant burden, only when restricting to missense-constrained genes did *NOTCH1* achieve a significant Bonferroni-corrected *p*-value (0.0044). Burden significance for both *FLT4* and *NOTCH1* was highly specific to CHD compared to an unrelated schizophrenia sample and was further confirmed by the gnomAD singleton comparison analysis. Ultra-rare variants driving these results were found only in individuals with TOF. The results are consistent with previous genome-wide significant findings for TOF from independent multi-center exome sequencing studies: for *FLT4* in two reports (Jin et al., 2017; Page et al., 2019), and for *NOTCH1* in one report (Page et al., 2019), analyzed using different approaches. These studies thus serve to help validate our burden test methodology, and provide important independent replication, further cementing these genetic findings for TOF, replicating *NOTCH1* for the first time, and collectively supporting study designs that focus on TOF within the heterogeneous umbrella of CHD.

For truncating variants, restricting to constrained genes did not result in identifying any other significant genes besides FLT4, even when considering a relatively inclusive significance threshold of BH-FDR < 10%. Including all genes with ultra-rare variants resulted in one other gene that passed BH-FDR < 10%, *CLDN9* (Claudin 9). *CLDN9* burden was not, however, confirmed by comparison to gnomAD singleton variants and the gene lacks evidence for involvement in cardiovascular development, thus at present we consider this result to be likely artifactual. Overall, our study results suggest that to limit such artifacts, considering only genes constrained for truncating variants may be especially important when a well-matched comparison data-set (here, schizophrenia WGS) is not available. For example, artifacts can arise if *de novo* mutation probabilities are derived from WGS data that were processed differently than the data available for the case-only cohort (e.g., different variant calling pipeline, QC filters, and principal transcripts). Also, denovolyzeR probabilities were generated for exome analyses and adjusted for sequencing depth, thus artifacts may arise in WGS studies where sequencing depth is greater. For missense variants, testing only genes passing a missense constraint threshold was less clearly beneficial. This is perhaps because missense constraint tends to be a characteristic of specific protein regions rather than the full gene product and this is not adequately modeled by gnomAD constraint indices.

For ultra-rare missense variants other than those in *NOTCH1*, we identified additional genes that were significant (BH-FDR < 10%) for the binomial test but not when comparing to gnomAD singletons (*BCKDK*, *DHH*, *KL*, *PRRT4*, *VMAC*, *KIAA0825*, *APC2*, *PXDN*). *BCKDK* (Branched-chain keto acid dehydrogenase kinase) is a negative regulator of the branched-chain amino acids catabolic pathways. *BCKDK* loss of function causes mainly neurological/neurobehavioral abnormalities (Joshi et al., 2006) in mice and humans (Novarino et al., 2012). Alterations of branched-chain amino acid metabolism have been described in relation to heart failure (Sun et al., 2016), however, there is no evident link between *BCKDK* and CHD. *DHH* (Desert hedgehog signaling molecule) is required for Sertoli cell and peripheral nerve development in mice, with mutations causing somewhat similar phenotypes in mice and humans (Bitgood et al., 1996; Parmantier et al., 1999; Umehara et al., 2000; Canto et al., 2004). No cardiac anomalies were reported, however, *DHH* was proposed to contribute to promoting ischemia-induced angiogenesis through a peripheral nerve mechanism (Renault et al., 2013). In humans, *KL* (Klotho) was previously proposed as a candidate gene for TOF because of overlapping ultra-rare loss CNVs at 13q13 (Costain et al., 2011, 2016). Deficiency of *Kl* in mice has profound systemic effects, with phenotype characterized by vascular calcification and atherosclerosis, reduced lifespan, cognitive impairment, stunted growth, skeletal abnormalities, and other organ alterations (Kuro-o et al., 1997). *Kl* is involved in the regulation of several pathways, including VEGF and Wnt (Mencke and Hillebrands, 2017). Considering this evidence, replication in larger cohorts and/or experimental data are required to conclusively implicate ultra-rare missense variants occurring in these gene in the etiology of CHD. Other genes had BH-FDR approaching 9% and were not reviewed in detail (*PRRT4*, *VMAC*, *KIAA0825*, *APC2*, *PXDN*).

### Functional Gene-Sets and Candidate Genes

Reassuringly, given our previously published results (Reuter et al., 2019), the gene-set burden analysis for truncating ultra-rare variants yielded a cluster corresponding to the VEGF pathway and blood vessel development (FDR = 0), and also a cluster corresponding to abnormal vasculature (FDR = 0.008). As expected (Reuter et al., 2019), *FLT4* was the main gene driving these results. We additionally identified other genes that were only nominally significant, but had suggestive functional or phenotypic evidence and could achieve genome-wide significance in a larger cohort.

Although we had previously identified some of these genes (*KDR* and *FOXO1*) (Reuter et al., 2019), *WNT5A* (Wnt family member 5A) and *ZFAND5* (zinc finger AN1-type containing 5) were identified only in this statistical re-analysis and appear as promising candidates for TOF/CHD. *ZFAND5* is transcriptionally activated by the platelet-derived growth factor (PDGF) pathway (Schmahl et al., 2007), and is reported to be a member of the FoxO family signaling pathway by the NCI-Nature PID pathway database. While heterozygous mice are apparently normal, mice homozygous for a *Zfand5* null mutation show loss of vascular smooth muscle cells that leads to widespread bleeding and postnatal death (Schmahl et al., 2007). *Wnt5a* loss disrupts second heart field cell deployment and other organ system development, and mice homozygous for a *Wnt5a* null allele die perinatally, with outflow tract defects (Yamaguchi et al., 1999; Schleiffarth et al., 2007; Sinha et al., 2015). *Wnt5a* also contributes to the vascular specification of cardiac progenitor cells and has a role in pressure overload-induced cardiac dysfunction (Reichman et al., 2018; Wang et al., 2019a). In humans, heterozygous missense or homozygous truncating variants in *WNT5A* are associated with multisystem ‘Robinow syndrome’ (OMIM: 180700) (Person et al., 2010; Birgmeier et al., 2018), with right ventricular outlet obstruction occurring as a relatively rare associated anomaly (Atalay et al., 1993).

The gene-set results also identified other constrained genes that support a role in the VEGF pathway or other complementary mechanisms for TOF. For truncating variants, these were from human (e.g., *AKAP12*), mouse (e.g., *EPN1*, *ATF2*) or both (*PKD1*) (Table 3) derived gene-sets (Fearnley et al., 2014; Tessneer et al., 2014; Benz et al., 2019; Villalobos et al., 2019; Wang et al., 2019b). Results for ultra-rare missense variants in *NOTCH1*, and from related gene-sets, may also support abnormal vascular development and related signaling as potential mechanisms in TOF.

We note that, collectively, ultra-rare variants in genes *FLT4*, *ZFAND5*, *WNT5A*, and *NOTCH1*, were present only in probands with TOF (i.e., not in the other-CHD subgroup), representing significant enrichment (Fisher’s exact test two-sided *p*-value = 0.01494) compared to background of the total sample. Also, individuals with ultra-rare variants in six key genes, truncating (*FLT4*, *KDR*, *FOXO1*, *ZFAND5, WNT5A*) or missense (*NOTCH1*), identified in this study correspond to 11.4% of those with TOF studied (*n* = 20/175; see Supplementary Table 14).

One may wonder why certain VEGF pathway genes that were previously implicated in TOF using manual curation of truncating variants in this data-set (Reuter et al., 2019) were not found in the gene-set analysis in the current study. There are several possible reasons. *BCAR1* was implicated by structural variation (thus not analyzed in the current study), *VEGFA* does not have a defined *de novo* mutation probability in denovolyzeR, *FGD5* and *PRDM1* are not associated to any VEGF-related gene-sets among the GO/pathways gene-sets used for this analysis, and *IQGAP1* was present only in a VEGF-related gene-set not containing *FLT4* and thus did not achieve significance. If these genes were also included, ultra-rare variants in the total 11 genes implicated would account for ∼14% of the adults with TOF in this study (*n* = 24/175).

It is worth noting that only one individual presented with a combination of ultra-rare risk variants of various types, and this involved a *NOTCH1* missense variant and a previously reported structural variant (thus not studied here) in a gene from the VEGF pathway (*BCAR1*) (Reuter et al., 2019). Assuming a plausible oligogenic model for TOF, one could expect, in addition to structural variants of all sizes including CNVs, that there would be contributions from other variant types not studied here, e.g., those of intermediate frequency, and/or ultra-rare non-coding variants. It is also likely that there are additional risk genes for TOF with ultra-rare variants that did not reach significance in the current study, and that would need an expanded cohort to be discovered. In addition to within individual (e.g., Supplementary Table S14) and between individual genetic, including allelic, heterogeneity, expected complexity includes variable clinical expression. The latter would include, e.g., the *NOTCH1* findings in TOF here and elsewhere (Page et al., 2019), expanding from initial association with a syndrome (AOS5). Also, a recent exome sequencing study of 49 patients with hypoplastic left heart syndrome reported rare truncating variants in *NOTCH1* as conveying significant risk for left ventricular outflow tract obstruction (Helle et al., 2019).

### Advantages and Limitations

Analyzing ultra-rare variant burden appears to be a suitable strategy, especially for TOF (Jin et al., 2017; Page et al., 2019; Reuter et al., 2019), given a genetic architecture characterized in a substantial minority by rare variants of large effect, though with reduced penetrance and likely oligogenic contributions. The method we adopted enables testing of ultra-rare genetic variant burden in a case-only cohort, without having access either to parents to determine variant *de novo* status, or to matched controls for case-control analysis. This would be a relatively common circumstance for many studies, especially of rare and under-funded conditions like TOF. In line with previous studies, we adopted a particularly stringent definition of ultra-rare variants, considering only variants observed in one CHD subject and never observed in gnomAD. Future studies leveraging larger and control-matched cohorts may identify additional contributing variants, and genes, by considering more prevalent rare variants (e.g., with allele frequency < 0.1%). This approach was not suitable, however, for the data-set available here and was thus not investigated.

In our study design, we attempted to address issues that can produce artifacts, such as mismatch of the variant calling, and/or processing pipelines, between those used for the disease data-set and for the data-set supporting the calculation of *de novo* probabilities. We had the advantage of access to a similarly sized sequencing data-set for an unrelated disease, processed in the same way, to aid in identifying potential artifacts that may not be available for future applications of this statistical burden method. Although we observed that restricting the burden analysis to genes constrained for truncating variants may help minimize such artifacts, advantages were less obvious using constraint for missense variants, and we note that the findings may be disease or study specific. As a further confirmatory analysis, we compared the ultra-rare burden in CHD to that in gnomAD. Additional analyses using a benchmark are required to establish whether one of these two methods is superior, in terms of power and minimizing artifacts. The advantage of using gnomAD singletons is that, while variant calling pipelines cannot be matched, other downstream processes like annotation can be matched to the disease data-set of interest.

All results were limited by the size of the cohort available with WGS data. Several gene-set clusters did not pass the multiple test correction yet appeared highly promising; an expanded cohort could reveal further significant findings. Like for all analyses using gene-sets, the lag in updating bioinformatics databases (such as GO and MPO) (Tomczak et al., 2018) constitutes a limitation. In addition, while the method identified highly relevant gene-set clusters for ultra-rare truncating variants, *FLT4* played a disproportionately large role in the analysis, likely influencing the fact that the relatively few novel candidate genes identified largely converged on the VEGF pathway. For other disorders that are even more genetically heterogeneous, the results suggest that optimizing the analysis method at the gene-set level may be essential in order to identify significant results (Marshall et al., 2017; Tomczak et al., 2018). As for all studies using statistical methods to identify potential disease candidate genes, additional experimental work would be required to conclusively implicate genes.

Future meta-analyses using this and other sequencing data-sets could reveal additional candidate genes with ultra-rare coding variants. Focusing on TOF appears particularly appealing, given that the most promising genes identified in this study had ultra-rare variants exclusively in the TOF subset, and that significant gene-sets results for ultra-rare missense variants were found only for TOF. Power analyses suggest that a sample of >900 TOF subjects would be required to achieve Bonferroni-corrected *p*-value < 0.05 for ultra-rare truncating variants in *KDR*, and an even larger sample size of >1,600 TOF subjects would be required for *FOXO1*, *WNT5A* and *ZFAND5*. Identifying other, more homogenous subsets within the broader CHD spectrum may also be beneficial.

Larger whole genome sequencing studies, ideally with matched controls, will be needed to study non-coding and structural variant burden. There are currently no published *de novo* mutation probability models for structural variants, and variability in variant calling pipelines would represent further major barriers to these analyses. Considering ultra-rare non-coding variants in regulatory elements like promoters and enhancers, a case-only cohort could be analyzed by leveraging singleton burden in the gnomAD v3 data-set (which comprises 71,702 whole genomes). While it would be ideal to strive for an analysis that could integrate ultra-rare variants of all variant types, and then less rare variants, the variability in genomic architecture between variant types will be amongst the challenges to overcome.

## Conclusion

The gene burden analysis method used, including a stringent Bonferroni correction, confirmed that genes *FLT4* with ultra-rare truncating variants, and *NOTCH1* with ultra-rare deleterious missense variants, are implicated in the etiology of TOF. The significant enrichment of *NOTCH1* missense variants in the extracellular domain, and specifically altering cysteine residues forming disulfide bonds, was also confirmed. Despite the small sample size, gene-set analysis identified ultra-rare truncating variants in novel candidate genes, including *ZFAND5* and *WNT5A*, as potentially implicated in the etiology of TOF. Other novel genes identified provide further confidence in the importance of the VEGF pathway to TOF. While several of these candidate genes are compelling, with supportive data from known functions and animal model phenotype, additional experimental work and/or replication in other data-sets are required to appreciate their potential role in the etiology and pathogenesis of TOF.

## Materials and Methods

### Study Participants and Genome Sequencing

This study was authorized by the Research Ethics Boards at the University Health Network (REB 98-E156)^4^, and centre for Addiction and Mental Health (REB 154/2002)^5^. Written consent was obtained from all participants or their legal guardians. We performed genome sequencing using DNA from 231 probands of European ancestry (175 TOF, 49 transposition of the great arteries, 7 other CHD) as previously described (Silversides et al., 2012; Costain et al., 2016; and Reuter et al., 2019) DNA was sequenced on the Illumina HiSeq X system^6^ at The Centre for Applied Genomics (TCAG)^7^. Libraries were amplified by PCR prior to sequencing. Libraries were assessed using Bioanalyzer DNA High Sensitivity chips and quantified by quantitative PCR using Kapa Library Quantification Illumina/ABI Prism Kit protocol (KAPA Biosystems). Validated libraries were pooled in equimolar quantities and paired-end sequenced on an Illumina HiSeq X platform following Illumina’s recommended protocol to generate paired-end reads of 150 bases in length.

### Variant Calling, Annotation, and Truncating and Missense Variant Extraction

#### Variant Calling

The paired FASTQ reads were mapped to the GRCh37 reference sequence using the BWA-backtrack algorithm (v0.7.12), and SNV and small indel variants were called using GATK (v3.7) according to GATK Best Practices recommendations (Depristo et al., 2011; Van der Auwera et al., 2013).

#### Variant Annotation

Variant calls were annotated using a custom pipeline based on ANNOVAR (July 2017 version) (Wang et al., 2010). Allele frequencies were derived from 1000 genomes (Aug. 2015 version) (Sudmant et al., 2015), ExAC (Nov. 2015 version) (Lek et al., 2016), and gnomAD (Mar. 2017 version) (Karczewski et al., 2019).

#### Classification of Variants by Truncating and Missense Effect

Truncating variants (labeled as *LOF* for *loss of function*) comprised frameshift insertions/deletions, alterations of the highly conserved intronic dinucleotide at splice sites and substitutions creating a premature stop codon (stop gain). Missense variants are substitutions of amino acids.

### Variant Filters Based on Quality, Allele Frequency and Effect

#### Allele Frequency Filter

The burden test adopted in this study was originally developed for *de novo* variants, but we argue that ultra-rare variants are not present in the general population and are likely to have arisen recently from *de novo* mutations transmitted to the progeny. We defined ultra-rare variants as appearing only once in the CHD WGS data-set and never in population reference data-sets (1000 genomes, ExAC, and gnomAD).

#### Low Quality Filter

We removed variants deemed to be low quality, which met at least one of these criteria: (i) low sequencing depth (DP ≤ 10); (ii) low alternate allele read fraction or low genotype quality (for heterozygous variants, alt_fraction < 0.3 or GQ ≤ 99, for homozygous variants, alt_fraction < 0.8 or GQ ≤ 25).

#### Frameshift Indel Filter

For each subject, whenever we found multiple indels on the same gene, we removed them from the variants list if their cumulative size was a multiple of 3. Otherwise, we kept one of the indels as a representative and removed the rest.

#### Splice Site Alteration Filter

For insertions overlapping splice sites, we considered them as truncating variants only if the alternate allele sequence did not encode a canonical AG/GT intronic dinucleotide.

#### Principal Transcript Effect Filter

We used the APPRIS database (assembly version: GRCh37, gene dataset: RefSeq105, Oct. 2018) to identify principal transcript isoforms (Rodriguez et al., 2018) and retained only variants with an effect on a principal transcript. APPRIS principal transcript identification is based on conservation, presence of protein domains and other coding sequence characteristics.

#### Final Ultra-Rare Variant Counts

We considered maximum only one ultra-rare missense or truncating variant per gene per subject, such that, for each variant type, the count of ultra-rare variants in a given gene equals the count of subjects with at least one variant in that given gene.

### Gene Burden Analysis

#### *De novo* Mutation Probabilities

We obtained *de novo* mutation probabilities for each gene from denovolyzeR^8^) (Ware et al., 2015). 1000 Genomes intergenic regions that are orthologous between humans and chimps were used to derive mutation probabilities. The probabilities were based on substitution type, trinucleotide context and other genome structure characteristics; in addition, they were adjusted for exome sequencing depth (Gibbs et al., 2014).

#### Rescale *de novo* Mutation Probability for Ultra-Rare Variants

Since the original mutation probabilities were estimated for *de novo* variants, we applied a multiplicative global scaling factor (SF), defined in equation [1], to obtain new rescaled probabilities *P*_exp,(LOF  or  Missense),g_; the scaling factor *SF* is computed so that the number of predicted and observed ultra-rare variants match.

(1) $$S\,F=\frac{N_{O\,b\,s,\left(L\,O\,F\,o\,r\,M\,i\,s\,s\,e\,n\,s\,e\right)}}{\sum_{g=1,\ldots ,G}\left(P_{e\,x\,p,\left(L\,O\,F\,o\,r\,M\,i\,s\,s\,e\,n\,s\,e\right),g}\right)\times N_{S}}$$

where *N*_Obs,(LOF  or  Missense)_ is the number of all observed truncating or missense ultra-rare variants; the denominator corresponds to the number of expected ultra-rare variants using the original unscaled probabilities: *G* is the total number of genes for which there is a defined mutation probability, including genes without any observed ultra-rare variant (in the analyses considering only constrained genes, note that *G* is further restricted to such genes); *G* is the expected *de novo* mutation probability for gene *P*_exp,(LOF  or  Missense),g_ with respect to truncating or missense variants; and *N*_S_ is the number of subjects in the study.

#### Binomial Test

Ultra-rare truncating and missense burden was tested using a one-sided binomial test comparing observed to expected rates, where expected rates correspond to the rescaled mutation probabilities. The alternative hypothesis is defined as P_success_ > N_success_/N_*trials*_, i.e., that the observed rate for a given gene exceeds the expected rate based on rescaled mutation probabilities.

(2) $$\left\{ \begin{array}{c} P_{S\,u\,c\,c\,e\,s\,s}=P_{e\,x\,p,\left(L\,O\,F\,o\,r\,M\,i\,s\,s\,e\,n\,s\,e\right),g}\times S\,F\;\\ N_{t\,r\,i\,a\,l\,s}=N_{S}\;\;\\ N_{S\,u\,c\,c\,e\,s\,s}=N_{O\,b\,s,\left(L\,O\,F\,o\,r\,M\,i\,s\,s\,e\,n\,s\,e\right),g}\; \end{array}\right.$$

where *N*_*Success*_ = *N*_*Obs*,(*LOForMissense*),*g*_ denotes the number of observed ultra-rare truncating or missense variants for gene *g*. Note that, for simplicity, we used ultra-rare variant counts in equation [2], but since we considered maximum only one ultra-rare truncating or missense variant per subject per gene, the truncating or missense variant count per gene is equivalent to the count of subjects with at least one truncating or missense ultra-rare variant in that gene.

#### gnomAD Comparison Analysis

SNVs and indels data were obtained from the gnomAD v2.1.1 database, comprising WES (125,748 subjects) and WGS (15,708 subjects), after restricting to the interval list (hg19-v0-wgs_evaluation_regions.v1.interval_list) used to generate the Exome Calling Intervals VCF file (gnomad.genomes.r2.1.1.exome_calling_intervals.sites.vcf.bgz). Genes were additionally restricted to have at least one truncating and at least one missense variant in gnomAD, in order to avoid genes that had been masked out by gnomAD (resulting in *n* = 17,304 genes). Singleton variants were identified by using the allele counts provided in the gnomAD VCF file and they were annotated using the same ANNOVAR-based pipeline, followed by the same effect filters as in the main analysis (including the selection of the same principal transcript) and finally categorized as truncating or missense. Genes were tested for burden by comparing CHD WGS ultra-rare variants to gnomAD singletons using a two-sided Fisher’s Exact Test, and specifically by constructing the 2 × 2 contingency matrix with counts: (a) CHD ultra-rare variants in the gene of interest, (b) CHD ultra-rare variants in other genes, (c) gnomAD singletons in the gene of interest, (d) gnomAD singletons in other genes; truncating and missense variants were tested separately. For CHD, only maximum one ultra-rare variant per subject was considered (as in the main analysis).

#### Multiple Test Correction

For gene burden analyses, multiple test correction was performed using the Benjamini-Hochberg False Discovery Rate (*BH-FDR*), as implemented in the R function *p.adjust*, and Bonferroni correction, by multiplying the *p*-value by the number of genes tested. For both corrections, we considered all genes with a defined probability, or all genes with a defined probability and passing constraint cut-offs (o/e gnomAD score < 0.35 for truncating variants and o/e gnomAD score < 0.75 for missense variants). For the Bonferroni correction, tests on truncating and missense variants were jointly considered. For the BH-FDR correction, they were considered separately. For the TOF-only analysis, we performed multiple test correction separately.

### Gene-Set Burden Analysis

#### Gene-Set Resources

GO/pathways gene-sets were derived from Gene Ontology (GO) annotations as provided by the Bioconductor package org.Hs.eg.db v3.5 (Carlson, 2019), BioCarta pathways^9^, KEGG pathways (see text footnote 2) retrieved using the KEGG API (Kanehisa et al., 2017), REACTOME pathways (Fabregat et al., 2018), and National Cancer Institute (NCI) pathways^10^. MPO gene-sets corresponding to phenotypes of mouse orthologs were derived from MPO gene annotations as provided by MGI (Bult et al., 2019).

#### Gene-Set Filters

We retained only the gene-sets with more than 5 genes and less than 100. Smaller gene-sets are detrimental for power. Larger gene-sets are usually removed because they are overly general. Considering the specific gene-level burden signal distribution observed for this data-set, characterized by the presence of two “highly concentrated” burden genes (*FLT4* and *NOTCH1*), some larger gene-sets could exceed the expected ultra-rare variant rate just because of the presence of one of these two genes. In addition, larger gene-sets are less suitable for the binomial test strategy, since they are more likely to present with more than one ultra-rare variant per subject and to contain genes with heterogeneous mutation probabilities, which is detrimental when pooling counts (Reimand et al., 2019).

For the analyses using a given gene constraint cut-off, we removed gene-sets with less than two genes passing the constraint cut-offs.

#### Binomial Test

For the gene-set analysis, we used a binomial test (equation [3]) to compare the number of observed and expected ultra-rare variants in the gene-set, similar to the gene burden analysis. We additionally ensured not to count more than one truncating or missense ultra-rare variant per gene-set per subject.

(3) $$\left\{ \begin{array}{c} P_{S\,u\,c\,c\,e\,s\,s}=\sum_{g\in G\,e\,n\,e\,S\,e\,t}P_{e\,x\,p,\left(L\,O\,F\,o\,r\,M\,i\,s\,s\,e\,n\,s\,e\right),g^{\times }}\,S\,F\;\\ N_{t\,r\,i\,a\,l\,s}=N_{S}\;\\ N_{S\,u\,c\,c\,e\,s\,s}=\sum_{s=1,\ldots ,S}m\,i\,n\,\left(\sum_{g\in G\,e\,n\,e\,S\,e\,t}N_{O\,b\,s,\left(L\,O\,F\,o\,r\,M\,i\,s\,s\,e\,n\,s\,e\right),g,s},1\right)\,\; \end{array}\right.$$

where *GeneSet* represents the set of all genes in a particular gene-set; *S* are the study subjects; and *N*_Obs,(LOF  or  Missense),g,s_ is the number of observed missense or truncating ultra-rare variants in a particular gene for subject *S*.

#### Greedy Step-Down Aggregation Method to Correct for Gene-Set Correlations

We addressed the problem of gene-set correlations, which are introduced by large gene overlaps between related gene-sets, by using a greedy step-down clustering approach, similar to what was adopted for highly correlated CNV locus gene testing in the *Marshall et al.* study (Marshall et al., 2017). The algorithm follows these steps, starting from an input list of gene-sets sorted by the ultra-rare burden binomial *p*-value[equation (4)]:

1. Select the gene-set with the most significant *p*-value (i.e., the smallest *p*-value);
2. Identify other gene-sets that are highly correlated to the selected gene-set, using the *Jaccard* similarity:

(4) $$\frac{\left|g\,s_{i}\,\cap g\,s_{j}\right|}{\left|g\,s_{i}\,\cup g\,s_{j}\right|}\;$$

where *gs*_i_ and *gs*_j_ are the sets of ultra-rare variants for gene-sets *i* and *j*, respectively. || is the number of ultra-rare variants in the corresponding set.

3. Cluster gene-sets that have *Jaccard* similarity >0.5 with the selected gene-set; these gene-sets will not be considered for the multiple test correction calculation, only the selected gene-set will be used (i.e., the *p*-value from the selected gene-set will be used as the *p*-value for the gene-set cluster). Finally, remove the selected gene-set and its clustered gene-sets from the sorted list.

### Resampling-Based FDR

Observed missense or truncating ultra-rare variants are resampled based on each gene’s rescaled mutation probability (equation [1]), while maintaining the same total number of observed missense or truncating ultra-rare variants. After this step, gene-sets are tested as described in the previous section. Finally, for each given *p*-value threshold *p*, the FDR is calculated as follows [equation (5)], considering only gene-sets selected by the greedy step-down aggregation procedure:

(5) $$F\,D\,R_{p}=\frac{m\,e\,a\,n_{i=1,\ldots ,1000}\,\left(N_{g\,s}^{p\,e\,r\,m\,u\,t\,a\,t\,i\,o\,n_{i}}\right)}{N_{g\,s}^{r\,e\,a\,l}}\;$$

where *FDR*_p_ is the FDR *q*-value for a given *p*-value threshold *p*, $N_{g\,s}^{\text{real}}$ is the number of gene-sets with binomial *p*-value ≤ p, and $N_{g\,s}^{p\,e\,r\,m\,u\,t\,a\,t\,i\,o\,n_{\text{i}}}$ corresponds to the number of gene-sets with binomial *p*-value ≤ *p* at iteration *i*. As stated in the formula, we used 1,000 sampling iterations.

### Power Analysis

Power analyses were performed using the function pwr.p.test from the R package pwr version 1.3-0.

## Data Availability Statement

All ultra-rare variants are included in the Supplementary Material. Complete variants files and whole genome read sequences are not publicly available because not all participants were consented for this purpose. Requests to access the datasets should be directed to ASB, anne.bassett@utoronto.ca.

## Ethics Statement

This study was authorized by the Research Ethics Boards at the University Health Network (REB 98-E156) (http://www.uhn.ca), and Centre for Addiction and Mental Health (REB 154/2002) (http://www.camh.ca). Written informed consent was obtained from all participants or their legal guardians.

## Author Summary

We analyzed the ultra-rare non-synonymous variant burden for genome sequencing data from 231 individuals with congenital heart defects, most with tetralogy of Fallot. We adapted a burden test originally developed for *de novo* variants. In line with other studies, we identified a significant truncating variant burden for *FLT4* and missense burden for *NOTCH1*. For *NOTCH1*, we observed frequent disruption of cysteine residues establishing disulfide bonds in the extracellular domain. We also identified genes with BH-FDR < 10% that were not previously implicated. To overcome limited power for individual genes, we tested gene-sets corresponding to functional pathways and mouse phenotypes. Gene-set burden of truncating variants was significant for vascular endothelial growth factor signaling and abnormal vasculature phenotypes. Burden in the most promising genes was mainly driven by the TOF subset. These results confirmed previous findings and suggested additional candidate genes for experimental validation in future studies. This methodology can be extended to other case-only sequencing data in which ultra-rare variants make a substantial contribution to genetic etiology.

## Author Contributions

RM and DM designed the statistical analysis framework. RM lead the data pre-processing and the statistical analysis. RM, DM, and ASB led the analysis interpretation and the manuscript writing. MSR contributed to analysis interpretation and to manuscript writing. WE contributed to the data pre-processing and to the statistical analysis. BAM, RC, MZ, RK, JBAO, EL, MC, RKCY, CRM, RKJ, and SWS contributed to analysis interpretation. TH, TN, and GP contributed to the data pre-processing. EO, RMW, and CKS contributed to patient recruitment and phenotype characterization. RHK and ASB coordinated the study and provided overall leadership. All authors discussed the results, provided critical feedback, and contributed to the final manuscript, and approved the submitted version.

## Conflict of Interest

DM is a shareholder of Deep Genomics Inc. The remaining authors declare that the research was conducted in the absence of any commercial or financial relationships that could be construed as a potential conflict of interest.

## Footnotes

1 http://cgap.nci.nih-.gov/Pathways/BioCarta_Pathways/

2 http://www.genome.jp/kegg/

3 http://pid.nci.nih.gov

4 http://www.uhn.ca

5 http://www.camh.ca

6 https://www.illumina.com/systems/sequencing-platforms/hiseq-x.html

7 http://www.tcag.ca

8 http://denovo-lyzer.org/

9 http://cgap.nci.nih.gov/Pathways/BioCarta-Pathways/

10 https://cactus.nci.nih-.gov/download/nci/

## Supplementary Material

FIGURE S1 Relation between gnomAD genetic constraint indices. **(A)** Relationship between pLI (*x* axis, discretized in three bins) and the ratio of observed/expected (o/e) truncating variants (*y* axis). pLI > 0.9 has often been used as haploinsufficiency cutoff for clinical variant interpretation, and gnomAD suggests using the upper bound of the o/e confidence interval < 0.35 for a similar use. We preferred using a point estimate <0.35 to be more inclusive, i.e., including genes with more moderate haploinsufficiency. For our analysis, we have considered genes with o/e score < 0.35. **(B)** Relationship between the missense constraint z-score (*x* axis, discretized in two bins) and the ratio of observed/expected missense variants (*y* axis). For our analysis, we have considered genes with o/e score < 0.75, which roughly corresponds to a *z*-score > 2, which in turn corresponds to a constraint *p*-value of 0.02275.

FIGURE S2 QQ-plots and *p*-value CHD/SZ scatterplot for the gene burden analysis restricted to constrained genes. **(A,C)** show the quantile-quantile (QQ) plots for gene burden *p*-values obtained for truncating ultra-rare variants restricted to constrained genes (gnomAD o/e < 0.35, **A**) or missense ultra-rare variants restricted to constrained genes (gnomAD o/e < 0.75, **B**). Only a few genes present *p*-values deviating from the null distribution, suggesting absence of systematic *p*-value inflation. **(B,D)** show scatterplots of the nominal *p*-values obtained for the gene burden analysis of truncating or missense ultra-rare variants in constrained genes, comparing CHD (*y* axis) versus schizophrenia (*x* axis). The most significant genes for CHD are typically not significant for SZ, suggesting the absence of systematic confounders.

FIGURE S3 Relation between the number of ultra-rare truncating variants per gene in the CHD data-set and in gnomAD. The distribution (across genes) of the number of ultra-rare truncating variants per gene is shown as an overlaid boxplot and violin plot for singletons in gnomAD (*x* axis), stratified by the number of ultra-rare variants in the CHD data-set (*y* axis); each dot represents a gene. The dashed line represents the linear regression predictions, which appear unreliable because of outliers and the small number of unique CHD ultra-rare variants counts. Only *FLT4* has 7 truncating ultra-rare variants, but the trend for other strata suggests that this is in large excess of singletons observed in gnomAD. Note that CHD ultra-rare variants are not observed in gnomAD, whereas gnomAD singletons are observed only once in gnomAD.

FIGURE S4 Relation between the number of ultra-rare missense variants per gene in the CHD data-set and in gnomAD. The distribution (across genes) of the number of ultra-rare missense variants per gene is shown as an overlaid boxplot and violin plot for singletons in gnomAD (*x* axis), stratified by the number of ultra-rare variants in the CHD data-set (*y* axis); each dot represents a gene. The dashed line represents the linear regression predictions, which appear robust. *KL* and *DHH* overlap with the lowest percentiles of the distribution, whereas *BCKDK* is lower than any observed value; only NOTCH1 has 8 missense ultra-rare variants, but the trend for other strata suggests that this is in excess of singletons observed in gnomAD. Note that CHD ultra-rare variants are not observed in gnomAD, whereas gnomAD singletons are observed only once in gnomAD.

FIGURE S5 Cytoscape enrichment map for the gene-sets with significant burden of ultra-rare truncating variants in constrained genes. An enrichment map visualizes gene-sets as a network based on their overlaps. Nodes correspond to gene-sets from the gene-set cluster with significant (FDR < 10%) burden for truncating ultra-rare variants in constrained genes, and edges correspond to the degree of overlap between gene-sets. Nodes are colored based on the burden nominal *p*-value, with darker red corresponding to more significant gene-sets. Edge thickness is proportional to the jaccard index obtained by considering ultra-rare truncating variants as set elements; only edges corresponding to jaccard index > 0.5 are displayed. Gene-set sub-clusters are suggested by automated network layout. Gene Ontology and pathways **(A)** are shown separately from mouse phenotypes **(B)**.

FIGURE S6 Power curves show the power calculations for passing a Bonferroni-corrected *p*-value of 0.05. **(A)** *KDR*, *FOXO1*, *WNT5A*, and *ZFAND5* for truncating variants; **(B)** *BCKDK*, *DHH*, and *KL* for missense variants. The *y*-axis is the power from 0 to 1, and the *x*-axis is the effect size of samples.

TABLE S1 Ultra-rare variants observed for 231 CHD samples including gnomAD o/e constraint scores.

TABLE S2 Gene burden statistics for CHD ultra-rare truncating variants.

TABLE S3 Gene burden statistics for CHD ultra-rare missense variants.

TABLE S4 Truncating variants observed in both SZ and CHD cohorts.

TABLE S5 Gene burden statistics obtained by comparing CHD ultra-rare variants to gnomAD singletons and tested using Fisher’s Exact Test.

TABLE S6 Missense Variants observed in both SZ and CHD cohorts.

TABLE S7 Gene-set burden statistics for ultra-rare truncating variants in clustered GO/pathways gene-sets.

TABLE S8 Gene-set burden statistics for ultra-rare truncating variants in GO/pathways gene-sets without clustering.

TABLE S9 Burden statistics for mouse phenotype gene-set clusters and ultra-rare truncating variants in constrained genes.

TABLE S10 Burden statistics for mouse phenotype gene-sets and ultra-rare truncating variants in constrained genes.

TABLE S11 Burden statistics for GO/pathways gene-set clusters and ultra-rare missense variants in constrained genes.

TABLE S12 Burden statistics for MPO gene-sets and ultra-rare missense variants in constrained genes.

TABLE S13 NOTCH1, BCKDK, DHH and KL missense variants details.

TABLE S14 Details of phenotype and additional rare variants in 20 adults with TOF and selected deleterious missense and truncating variants.

TABLE S15 Gene burden statistics for TOF-only ultra-rare truncating and missense variants.

TABLE S16 Gene-set burden statistics for TOF-only ultra-rare truncating and missense variants in clustered GO/pathways and mouse phenotype gene-sets.
