# Supplementary figures and images for: Genes and Pathways Implicated in Tetralogy of Fallot Revealed by Ultra-Rare Variant Burden Analysis in 231 Genome Sequences
Source: Front Genet. 2020 Sep 15;11:957. doi: 10.3389/fgene.2020.00957 (PMC7522597; doi:10.3389/fgene.2020.00957)

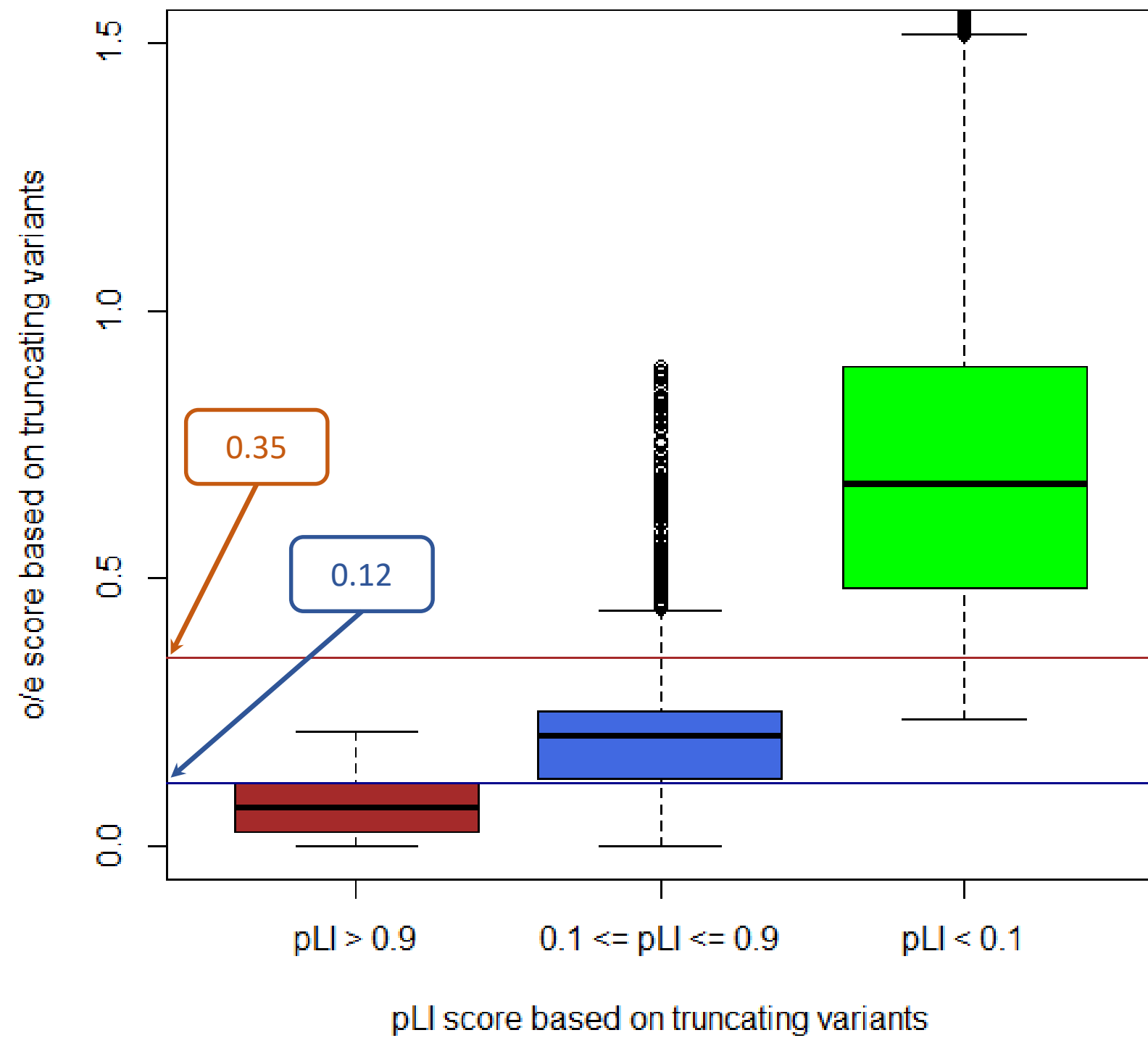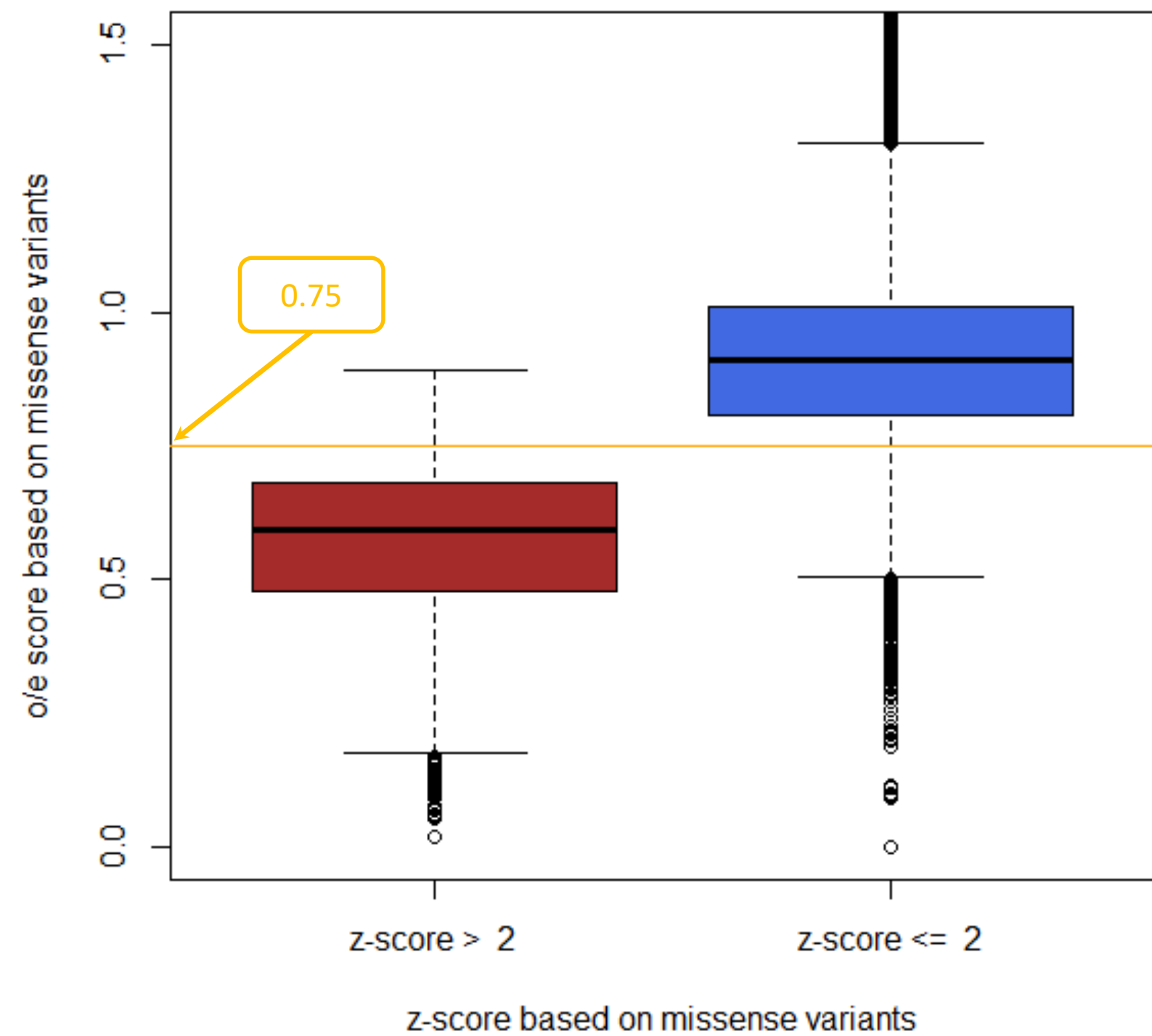

Supplement: FIGURE S1 — Relation between gnomAD genetic constraint indices. (A) Relationship between pLI (x axis, discretized in three bins) and the ratio of observed/expected (o/e) truncating variants (y axis). pLI > 0.9 has often been used as haploinsufficiency cutoff for clinical variant interpretation, and gnomAD suggests using the upper bound of the o/e confidence interval < 0.35 for a similar use. We preferred using a point estimate <0.35 to be more inclusive, i.e., including genes with more moderate haploinsufficiency. For our analysis, we have considered genes with o/e score < 0.35. (B) Relationship between the missense constraint z-score (x axis, discretized in two bins) and the ratio of observed/expected missense variants (y axis). For our analysis, we have considered genes with o/e score < 0.75, which roughly corresponds to a z-score > 2, which in turn corresponds to a constraint p-value of 0.02275. [file Data_Sheet_1.zip › SupplementaryFigures_FrontiersInGenetics/Supplementary Figure S1) Relation between gnomAD genetic constraint indexes.PDF]

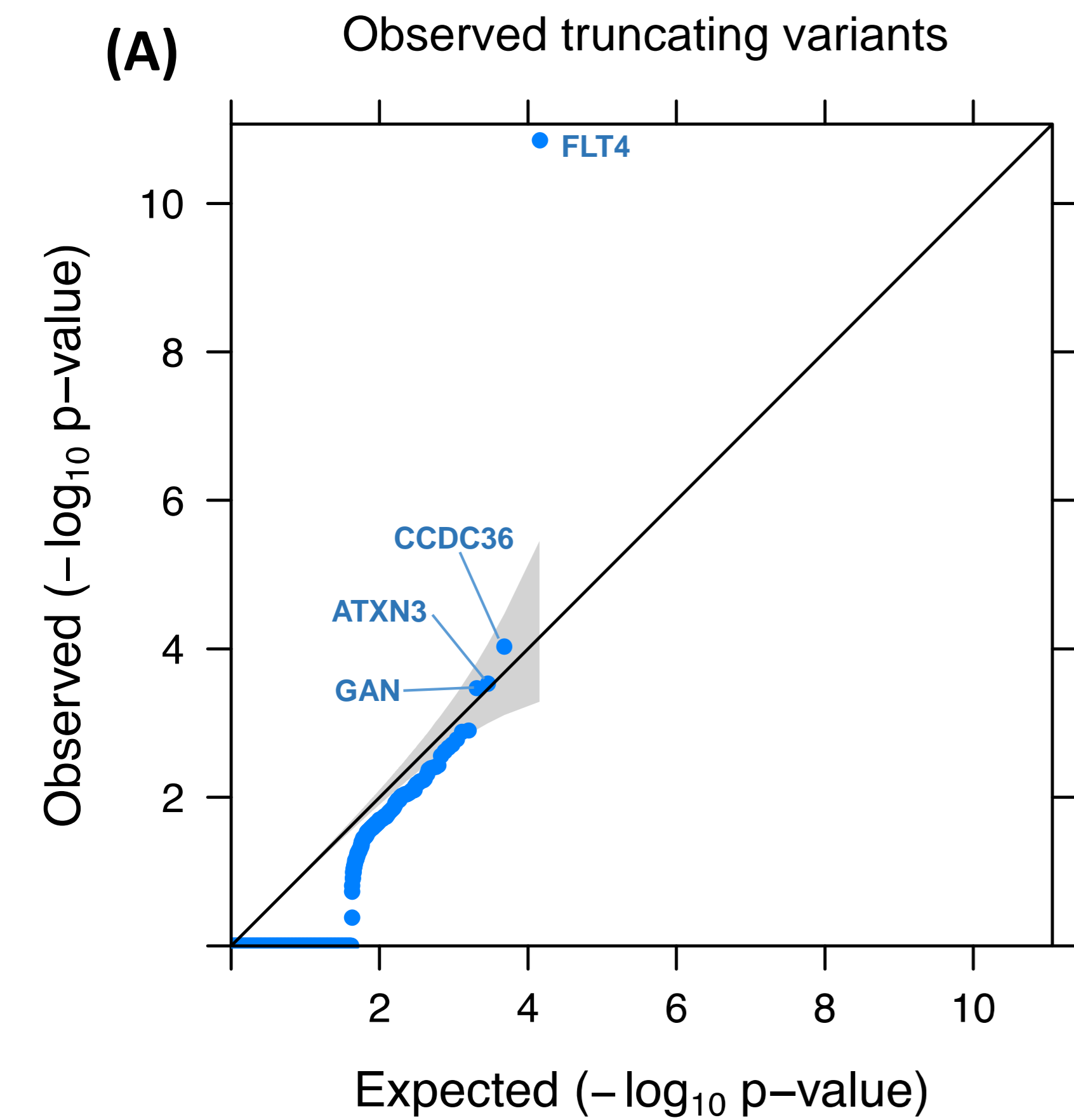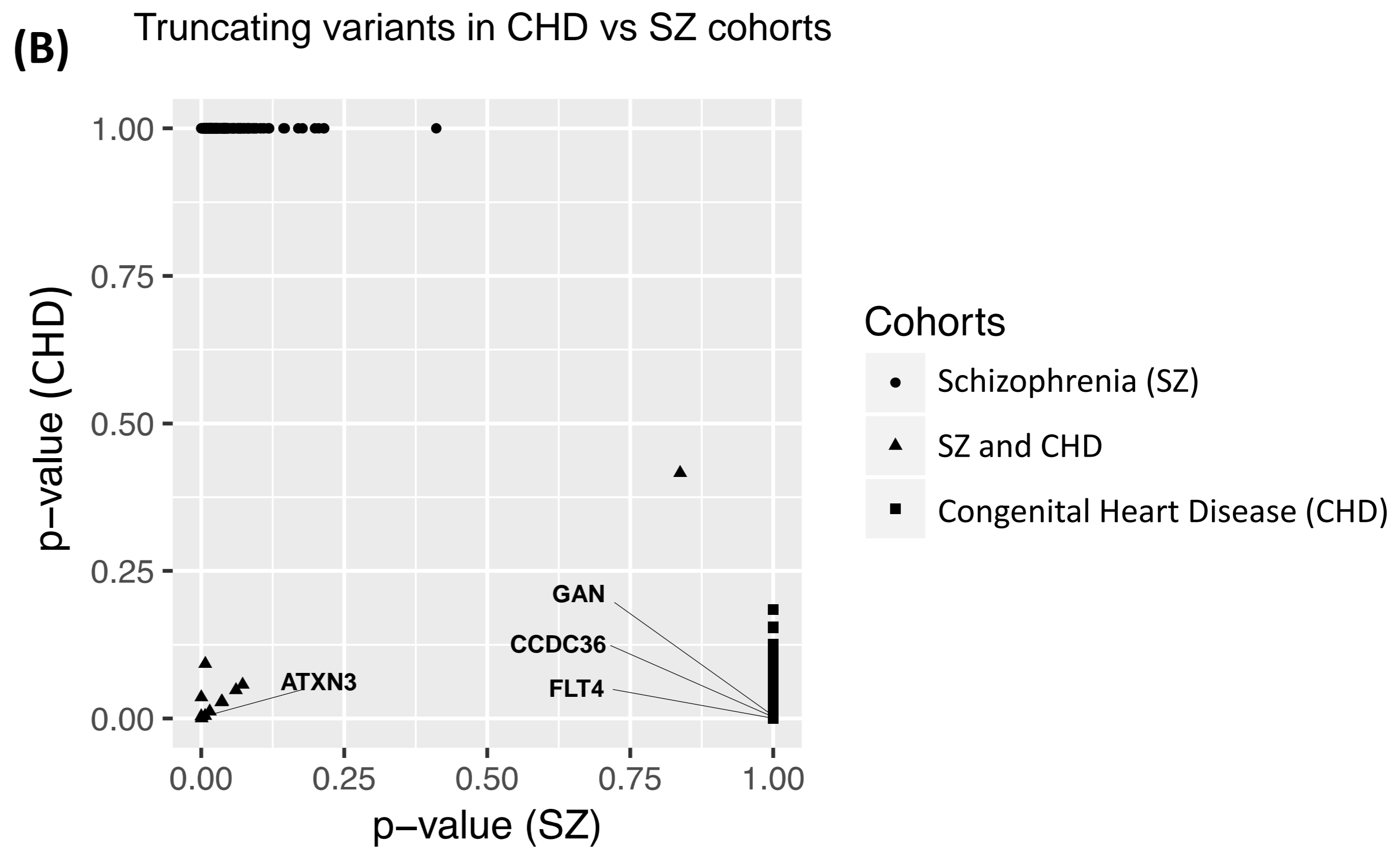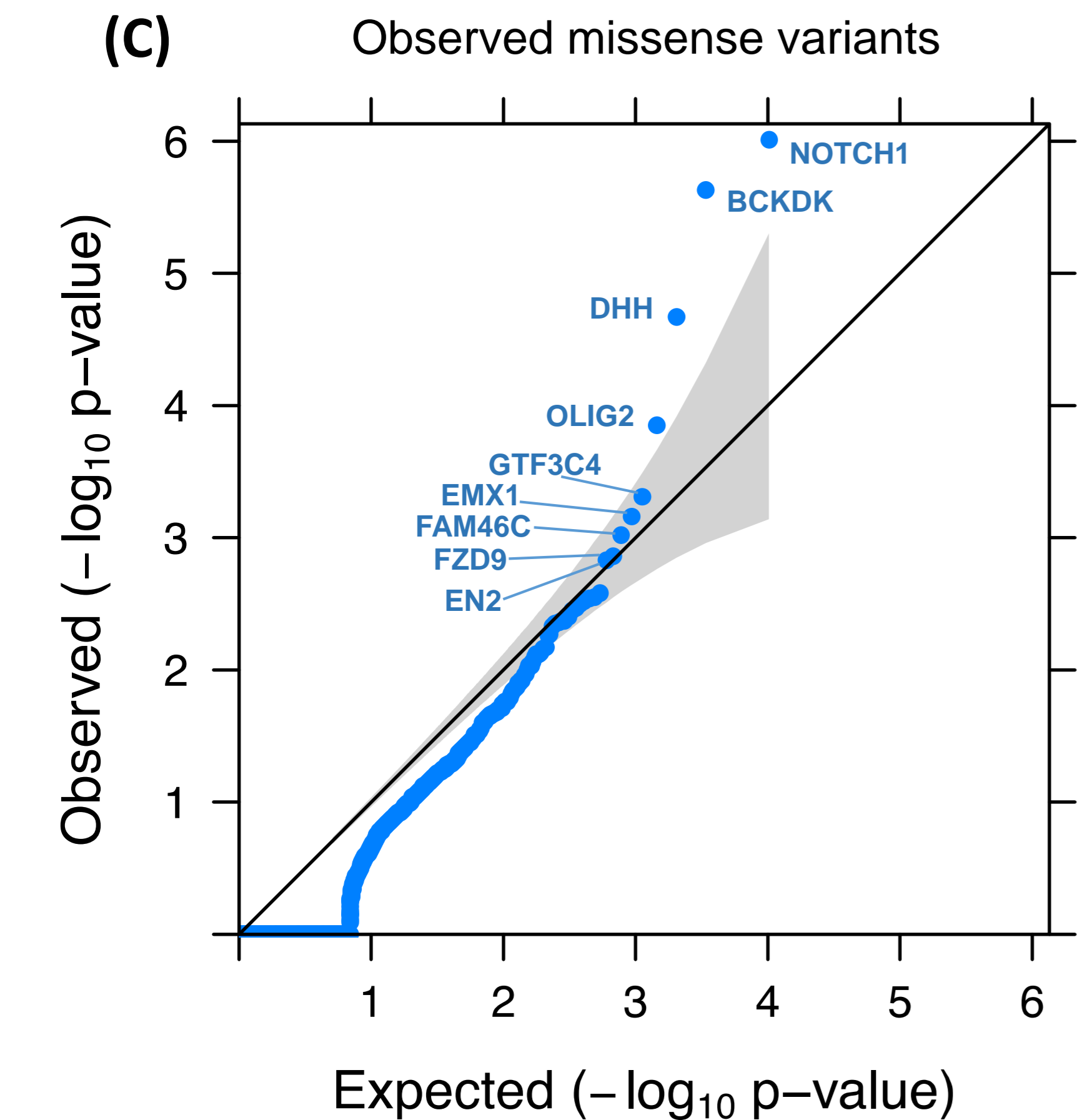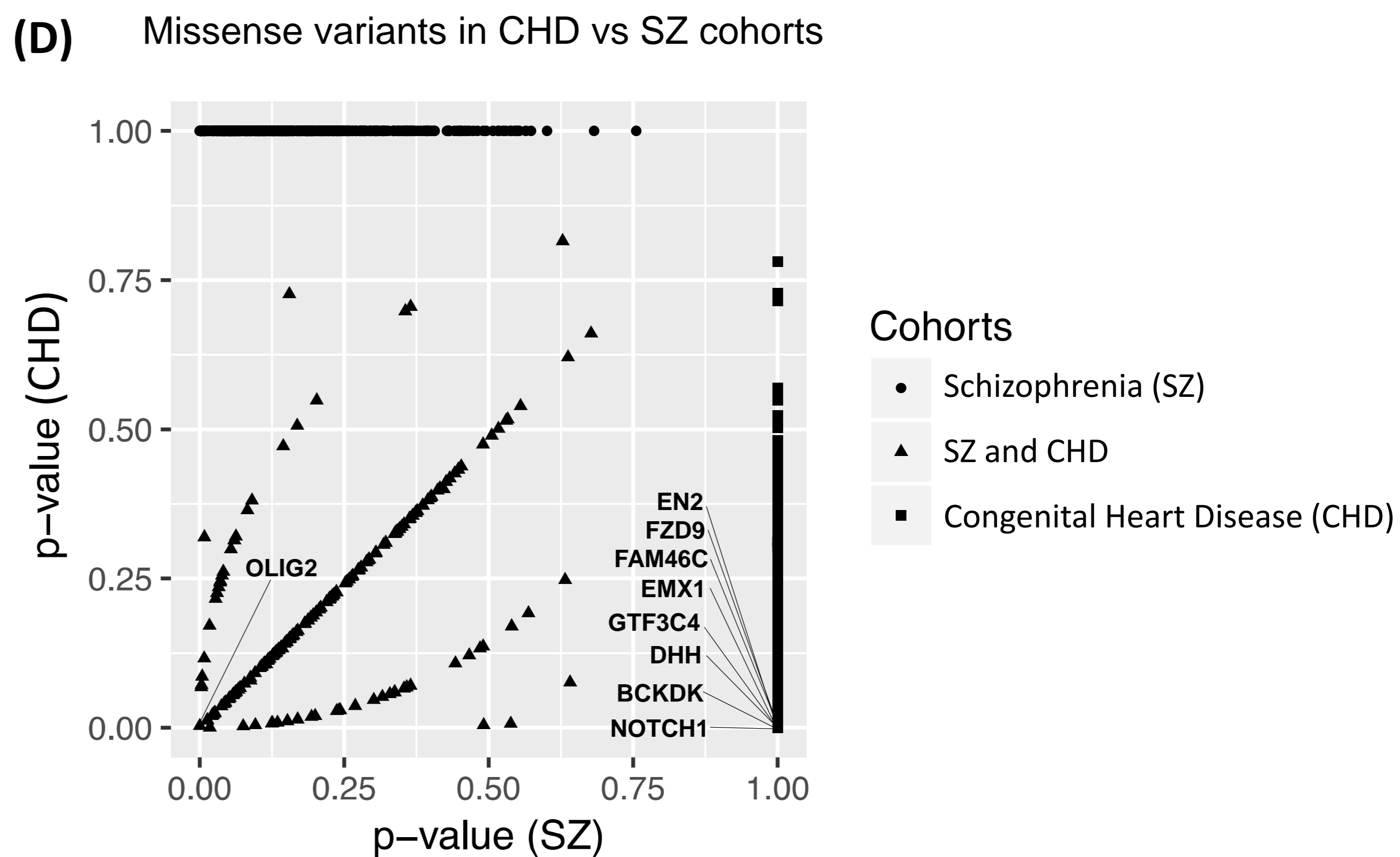

Supplement: FIGURE S1 — Relation between gnomAD genetic constraint indices. (A) Relationship between pLI (x axis, discretized in three bins) and the ratio of observed/expected (o/e) truncating variants (y axis). pLI > 0.9 has often been used as haploinsufficiency cutoff for clinical variant interpretation, and gnomAD suggests using the upper bound of the o/e confidence interval < 0.35 for a similar use. We preferred using a point estimate <0.35 to be more inclusive, i.e., including genes with more moderate haploinsufficiency. For our analysis, we have considered genes with o/e score < 0.35. (B) Relationship between the missense constraint z-score (x axis, discretized in two bins) and the ratio of observed/expected missense variants (y axis). For our analysis, we have considered genes with o/e score < 0.75, which roughly corresponds to a z-score > 2, which in turn corresponds to a constraint p-value of 0.02275. [file Data_Sheet_1.zip › SupplementaryFigures_FrontiersInGenetics/Supplementary Figure S2) QQ-plots and p-value CHD:SZ scatterplot for the gene burden analysis restricted to constrained genes.PDF]

based on truncating variants

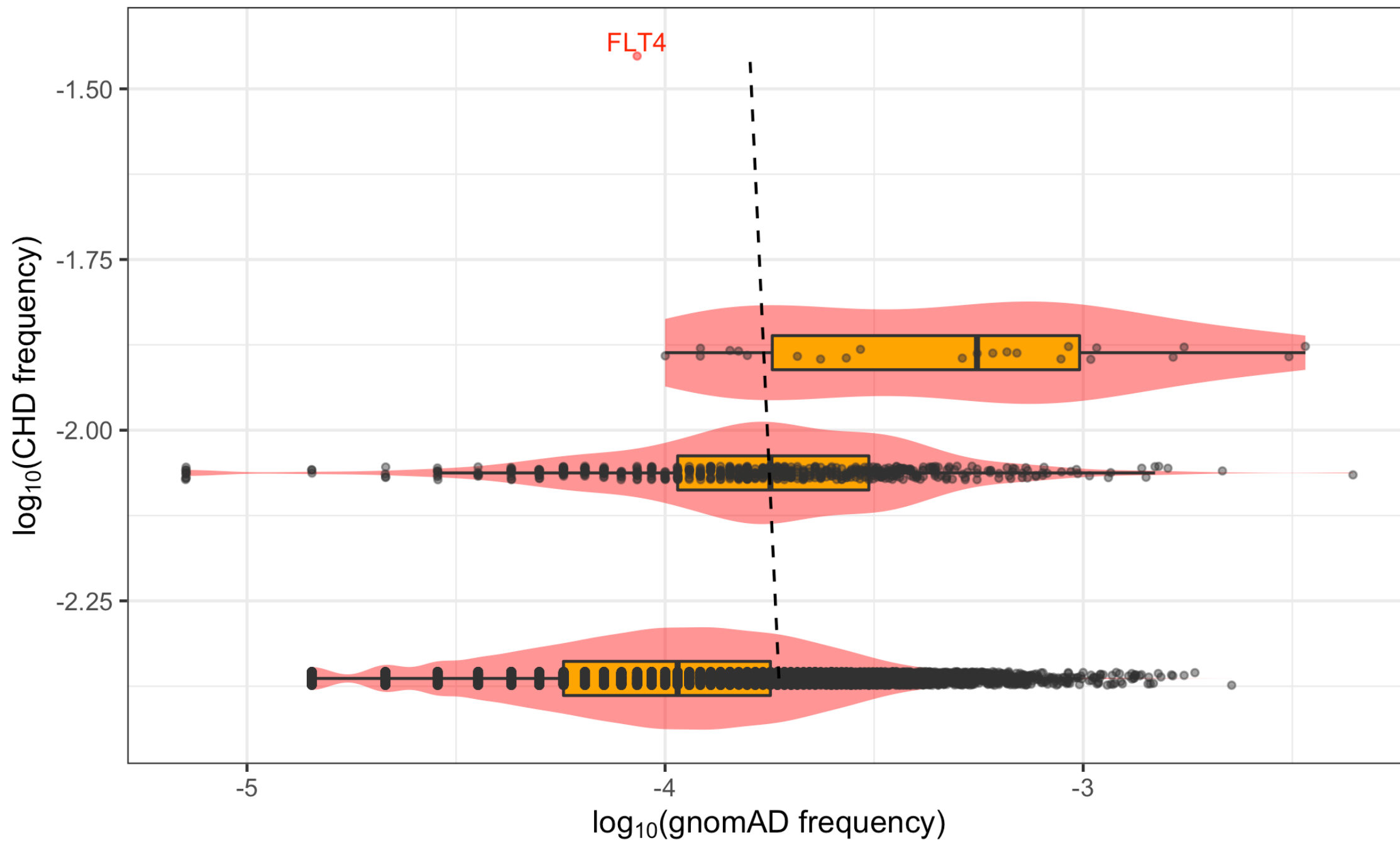

Supplement: FIGURE S1 — Relation between gnomAD genetic constraint indices. (A) Relationship between pLI (x axis, discretized in three bins) and the ratio of observed/expected (o/e) truncating variants (y axis). pLI > 0.9 has often been used as haploinsufficiency cutoff for clinical variant interpretation, and gnomAD suggests using the upper bound of the o/e confidence interval < 0.35 for a similar use. We preferred using a point estimate <0.35 to be more inclusive, i.e., including genes with more moderate haploinsufficiency. For our analysis, we have considered genes with o/e score < 0.35. (B) Relationship between the missense constraint z-score (x axis, discretized in two bins) and the ratio of observed/expected missense variants (y axis). For our analysis, we have considered genes with o/e score < 0.75, which roughly corresponds to a z-score > 2, which in turn corresponds to a constraint p-value of 0.02275. [file Data_Sheet_1.zip › SupplementaryFigures_FrontiersInGenetics/Supplementary Figure S3) Relation between the number of ultra-rare truncating variants per gene in the CHD data-set and in gnomAD.PDF]

based on missense variants

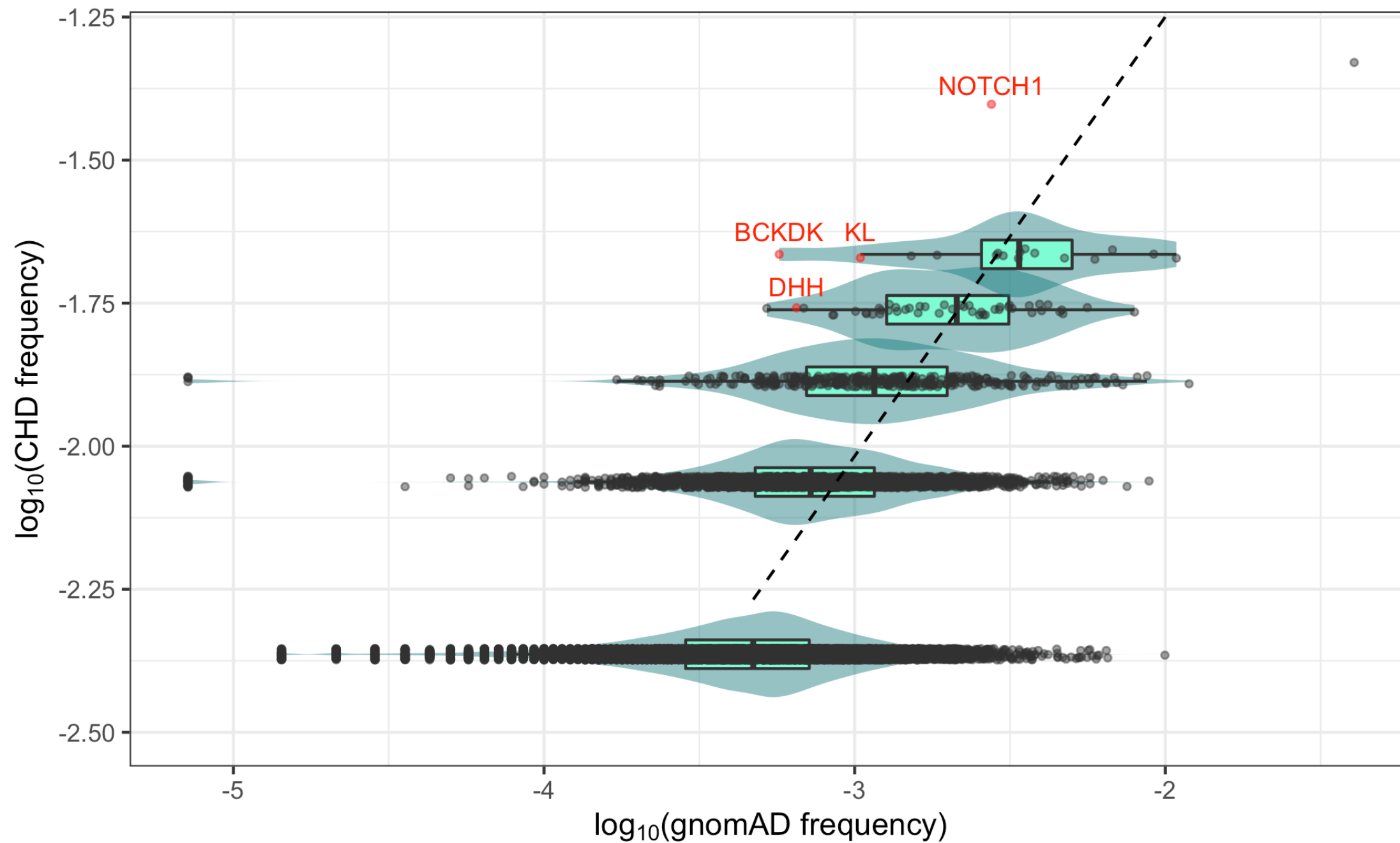

Supplement: FIGURE S1 — Relation between gnomAD genetic constraint indices. (A) Relationship between pLI (x axis, discretized in three bins) and the ratio of observed/expected (o/e) truncating variants (y axis). pLI > 0.9 has often been used as haploinsufficiency cutoff for clinical variant interpretation, and gnomAD suggests using the upper bound of the o/e confidence interval < 0.35 for a similar use. We preferred using a point estimate <0.35 to be more inclusive, i.e., including genes with more moderate haploinsufficiency. For our analysis, we have considered genes with o/e score < 0.35. (B) Relationship between the missense constraint z-score (x axis, discretized in two bins) and the ratio of observed/expected missense variants (y axis). For our analysis, we have considered genes with o/e score < 0.75, which roughly corresponds to a z-score > 2, which in turn corresponds to a constraint p-value of 0.02275. [file Data_Sheet_1.zip › SupplementaryFigures_FrontiersInGenetics/Supplementary Figure S4) Relation between the number of ultra-rare missense variants per gene in the CHD data-set and in gnomAD.PDF]

GO, NCI,  
BIOC, KEGG,  
REACT

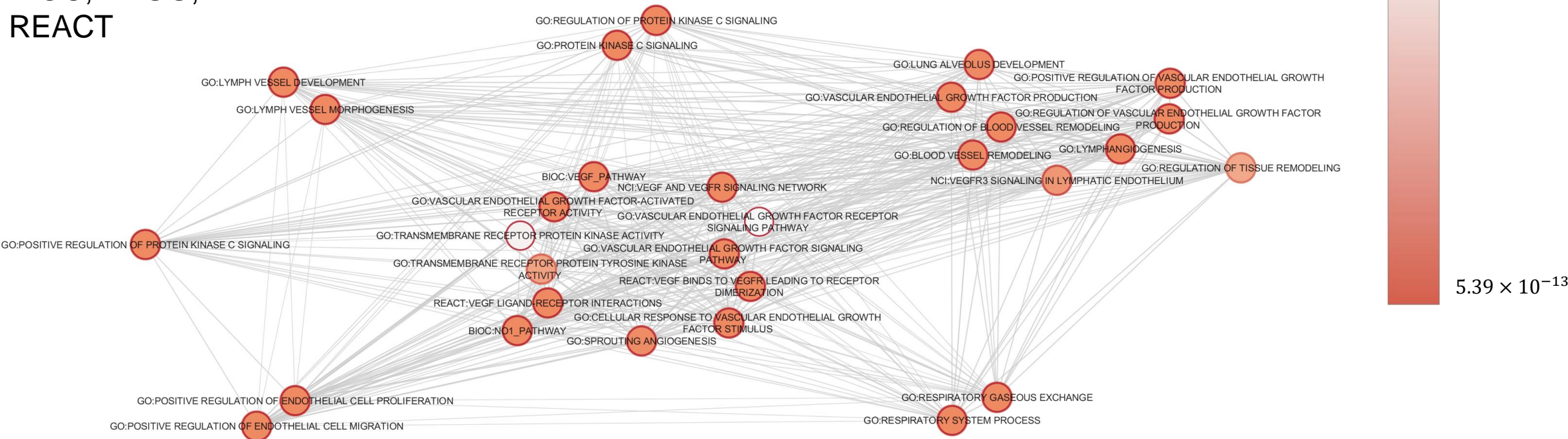

MPO

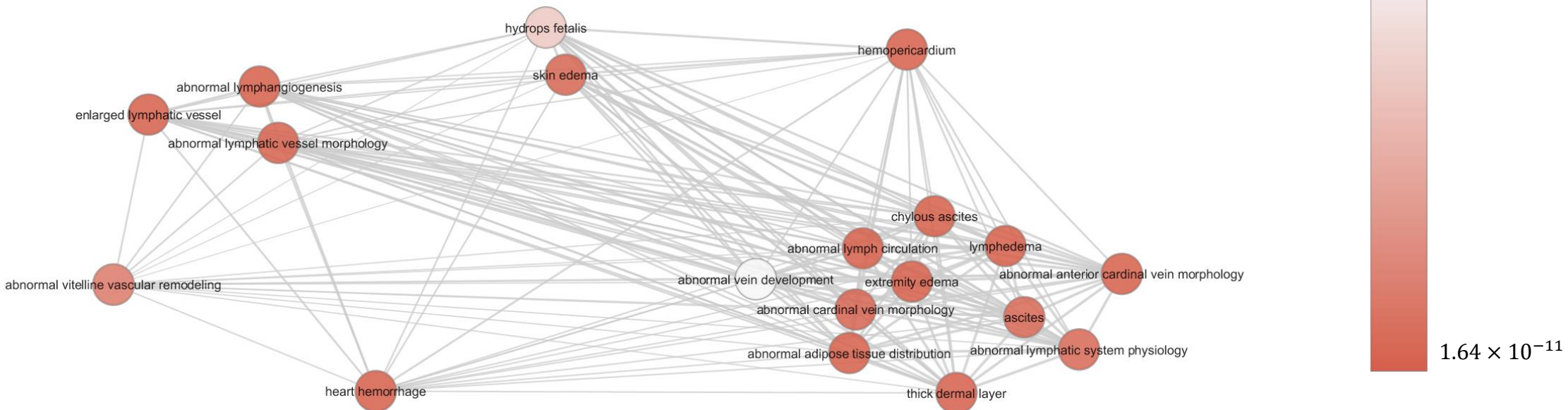

Supplement: FIGURE S1 — Relation between gnomAD genetic constraint indices. (A) Relationship between pLI (x axis, discretized in three bins) and the ratio of observed/expected (o/e) truncating variants (y axis). pLI > 0.9 has often been used as haploinsufficiency cutoff for clinical variant interpretation, and gnomAD suggests using the upper bound of the o/e confidence interval < 0.35 for a similar use. We preferred using a point estimate <0.35 to be more inclusive, i.e., including genes with more moderate haploinsufficiency. For our analysis, we have considered genes with o/e score < 0.35. (B) Relationship between the missense constraint z-score (x axis, discretized in two bins) and the ratio of observed/expected missense variants (y axis). For our analysis, we have considered genes with o/e score < 0.75, which roughly corresponds to a z-score > 2, which in turn corresponds to a constraint p-value of 0.02275. [file Data_Sheet_1.zip › SupplementaryFigures_FrontiersInGenetics/Supplementary Figure S5) Cytoscape enrichment map for the gene-sets with significant burden of ultra-rare truncating variants in constrained genes.PDF]

**A** Effect sizes estimated from 175 TOF subjects

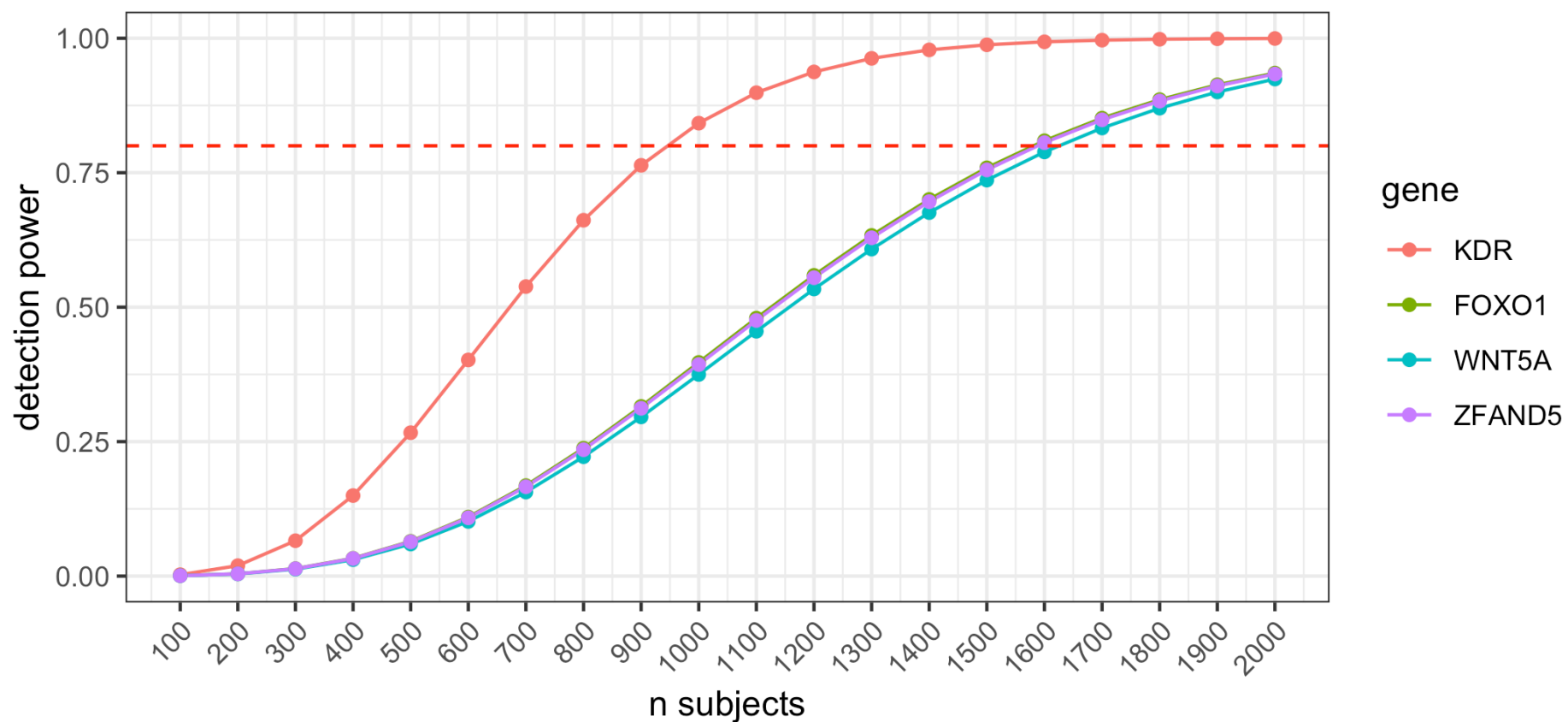

**B** Effect sizes estimated from 231 CHD subjects

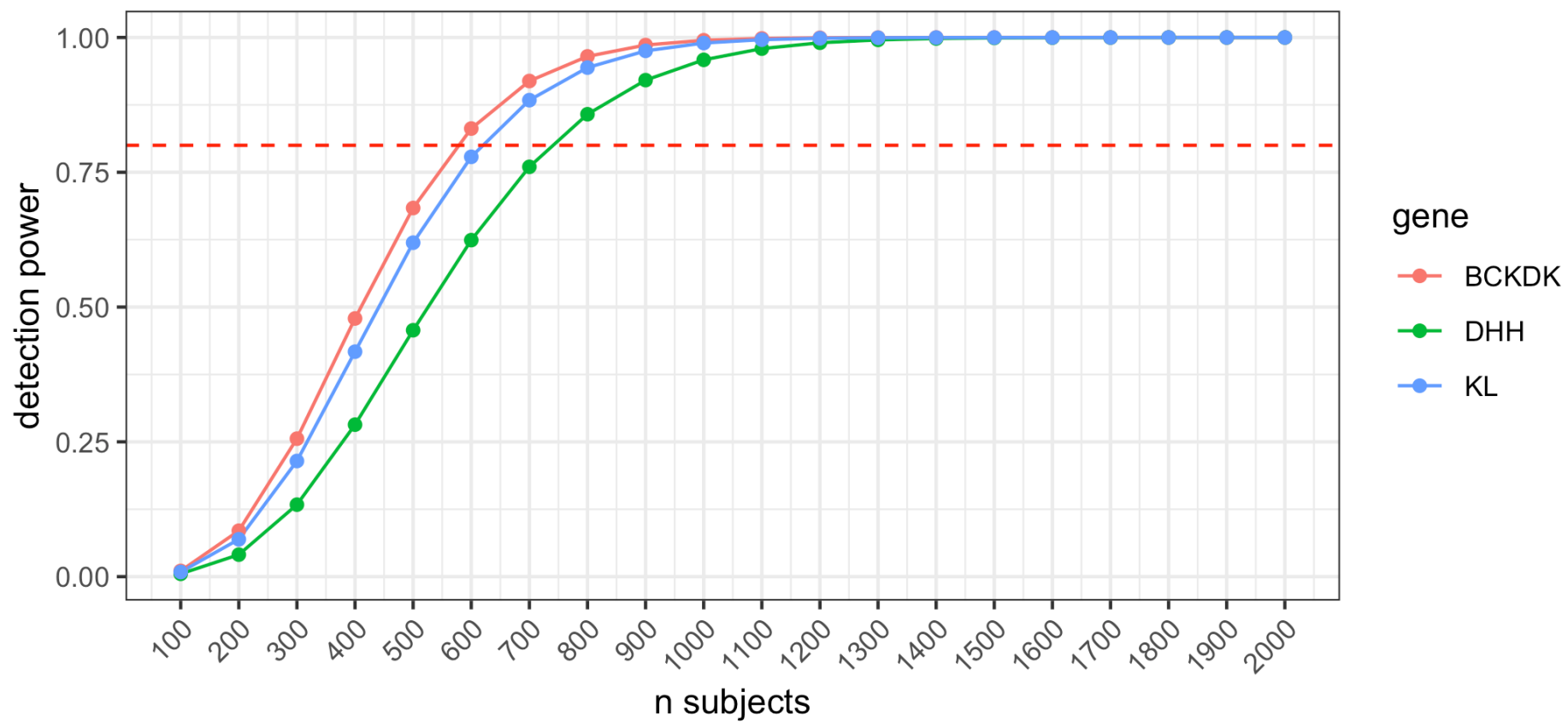

Supplement: FIGURE S1 — Relation between gnomAD genetic constraint indices. (A) Relationship between pLI (x axis, discretized in three bins) and the ratio of observed/expected (o/e) truncating variants (y axis). pLI > 0.9 has often been used as haploinsufficiency cutoff for clinical variant interpretation, and gnomAD suggests using the upper bound of the o/e confidence interval < 0.35 for a similar use. We preferred using a point estimate <0.35 to be more inclusive, i.e., including genes with more moderate haploinsufficiency. For our analysis, we have considered genes with o/e score < 0.35. (B) Relationship between the missense constraint z-score (x axis, discretized in two bins) and the ratio of observed/expected missense variants (y axis). For our analysis, we have considered genes with o/e score < 0.75, which roughly corresponds to a z-score > 2, which in turn corresponds to a constraint p-value of 0.02275. [file Data_Sheet_1.zip › SupplementaryFigures_FrontiersInGenetics/Supplementary Figure S6) Power curves show the power calculations for passing a Bonferroni-corrected p-value of 0.05.PDF]
