# Supplementary material for: Genes and Pathways Implicated in Tetralogy of Fallot Revealed by Ultra-Rare Variant Burden Analysis in 231 Genome Sequences
Source: Front Genet. 2020 Sep 15;11:957. doi: 10.3389/fgene.2020.00957 (PMC7522597; doi:10.3389/fgene.2020.00957)
Supplement: TABLE S1 — Ultra-rare variants observed for 231 CHD samples including gnomAD o/e constraint scores. [file Data_Sheet_2.zip › SupplementaryTables_FrontiersInGenetics/Supplementary Table S14) Details of phenotype and additional rare variants in 20 adults with TOF and selected deleterious missense and truncating variants.PDF]

Supplementary Table 14. Details of phenotype and additional rare variants in 20 adults with TOF and selected deleterious missense and truncating variants.

Case [a]	Phenotype and family history of CHD	Gene (transcript)	Variant [b]	Chromosomal position (GRCh37/hg19)	CNVs (and selected genes overlapped) [a]	Other putative risk variants [a]
**Deleterious missense variants (n=8 in *NOTCH1*), all but one (TOF220) not previously reported [a]**						
**TOF272**	TOF; VF and cardiac arrest age 39 y requiring ICD; hypothyroidism, died age 53 y; daughter with unspecified CHD	*NOTCH1* (NM_017617.3)	c.847T>G, p.(Cys283Gly)	chr9:139413913A>C	--	--
**TOF132**	TOF; endocarditis, depression, died age 59 y; infant daughter with TOF, persistent left SVC, solitary right thyroid lobe, Meckel's diverticulum, ectopic pancreas, died post-operatively	*NOTCH1* (NM_017617.3)	c.1243G>A, p.(Glu415Lys)	chr9:139412601C>T	--	--
**TOF310**	TOF; asthma; sister with unspecified CHD, died at 3 days	*NOTCH1* (NM_017617.3)	c.1816G>A, p.(Glu606Lys)	chr9:139410022C>T	10 kb deletion, chr7: 70197277-70207851, *AUTS2*	--
**TOF220**	TOF, RAA, APV; learning difficulties	*NOTCH1* (NM_017617.3)	c.1869C>A, p.(Asn623Lys)	chr9:139409969G>T	64 kb (exons2-7) deletion, chr16:75237177-75301117, BCAR1 (VEGF pathway gene, Reuter et al., 2019)<br>337 kb deletion, chr7:11374706-11711740, *THSD7A,* exons 2-28	--
**TOF303**	TOF; depression	*NOTCH1* (NM_017617.3)	c.2045G>A, p.(Cys682Tyr)	chr9:139409124C>T	341 kb duplication, chr15: 32458661-32799827, *GOLGA8O*, *CHRNA7*	--
**TOF174**	TOF; Hageman factor XII deficiency	*NOTCH1* (NM_017617.3)	c.2128G>A, p.(Asp710Asn)	chr9:139409041C>T	--	--
**TOF57**	TOF, PA, RAA; anxiety, depression	*NOTCH1* (NM_017617.3)	c.2444G>A, p.(Cys815Tyr)	chr9:139407496C>T	608 kb duplication, chr1: 120747707-121355881, *SRGAP2D*, *FAM72B*, *FCGR1B*;<br>15 kb deletion,	--

					chr5: 132918122-132933128, *FSTL4*, intronic	
**TOF131**	TOF, BAV; daughter with BAV and aortic coarctation, brother with hypoplastic left heart and aortic atresia, died at 9 days	*NOTCH1* (NM_017617.3)	c.4606T>C, p.(Cys1536Arg)	chr9:139399537A>G	265 kb deletion, chr10: 81293615-81559401, *NUTM2B*, *SFTPA2*, *SFTPA1*	--
**Truncating variants identified in the current study and not previously reported (n=3; n=1 each in *FLT4, WNT5A, ZFAND5*)**						
**TOF178[c]**	TOF, RAA, Late onset atrial flutter/fibrillation; depression	*FLT4* (NM_182925.4)	c.2766_2767dupCC, p.(Leu923Profs*4)	chr5:180046104dupGG	260 kb duplication, chr2: 98013814-98274527, *ANKRD36B*, *COX5B*, *ACTR1B*	--
**TOF133**	TOF, PA, RAA; anxiety, depression, died age 49 y	*WNT5A* (NM_003392.4)	c.486C>A, p.(Cys162*)	chr3:55508563G>T	--	--
**TOF120**	TOF, PA, RAA; strabismus, ADHD, intussusception, scoliosis	*ZFAND5* (NM_001278245.1)	c.337_340delACTA, p.(Thr113Profs*118)	chr9:g.74974361delTAGT	401 kb duplication, chr15: 32458661-32859858, *GOLGA8O*, *CHRNA7*	--
**Truncating variants previously reported (n=9; n=6 in *FLT4*, n=2 in *KDR*, n=1 in *FOXO1*)**						
**TOF158**	TOF, RAA, paroxysmal atrial flutter requiring ablation, mild aortic dilatation; depression and/or anxiety, migraine, melanoma; daughter with TOF shown to have inherited the same *FLT4* variant	*FLT4* (NM_182925.4)	c.3574C>T, p.(Gln1192*)	chr5:180038443G>A	--	*CHD4* (NM_001273.2): c.1475G>A, p.(Arg492His)
**TOF238**	TOF, RAA, MAPCA, PA; aortic dilatation	*FLT4* (NM_182925.4)	c.2499C>G, p.(Tyr833*)	chr5:180047216G>C	152 kb deletion, chr17: 47978986-48130937, *DLX4*, *DLX3*	*PLXND1* (NM_015103.2): c.2138C>T, p.(Ala713Val)
**TOF284**	TOF, MAPCA, inconclusive results about RAA; aortic valve replacement	*FLT4* (NM_182925.4)	c.1622dupG, p.(Gln542Profs*3)	chr5:180049766dupC	--	--
**TOF254**	TOF, APV; bilateral femoral vein occlusions; depression and/or anxiety	*FLT4* (NM_182925.4)	c.1172_1173delAG, p.(Glu391Glyfs*35)	chr5:180053196delCT	203 kb duplication, chr12: 45942271-46145500, *ARID2*, exons 1-3	--
**TOF68**	TOF, RAA, APV; depression and/or anxiety	*FLT4* (NM_182925.4)	c.1037delC, p.(Thr346Argfs*7)	chr5:180055948delG	24 kb duplication, chr19: 34210751-34234569, *CHST8*, intron 3	--

**TOF301**	TOF, RAA, paternal first cousin with suspected VSD	*FLT4* (NM_182925.4)	c.3331+1G>T, p.?	chr5:180041067C>A	--	*TLL1* (NM_012464.4): c.2996G>T, p.(Arg999Ile)
**TOF109**	TOF, PFO or ASD, atrial flutter; obesity; mild cognitive and memory problems attributed to cerebral ischemia; brother died in infancy of suspected cyanotic CHD	*KDR* (NM_002253.2)	c.3287G>A, p.(Trp1096*)	chr4:55955875C>T	--	--
**TOF155**	TOF, PFO or ASD; depression and/or anxiety; gastroesophageal reflux	*KDR* (NM_002253.2)	c.2638C>T, p.(Arg880*)	chr4:55962486G>A	129 kb deletion, chr7: 107653165-107782590, *LAMB4*	*RAF1* (NM_002880.3): c.1225A>G, p.(Met409Val)
**TOF62**	TOF, RAA; learning difficulties	*FOXO1* (NM_002015.3)	c.580_586delGTGCCCT, p.(Val194Thrfs*137)	chr13:41239764delAGGGCAC	78 kb duplication, chr8: 73708969-73787448, *KCNB2*	--

All variants are heterozygous. Subjects in this table are of European descent, by study design. Obesity was defined as body mass index (BMI) consistently >30 as an adult. Short stature was defined as height <3rd percentile using standard adult growth curves (see Reuter et al., 2019).

[a] Case numbers of participants are the same as those used in a previous report (Reuter et al., 2019) of loss-of-function variants affecting the VEGF pathway in tetralogy of Fallot (TOF). Phenotype, family history, and additional rare variants, including CNVs, are those previously reported in Tables 1, S3 and S6 in Reuter et al., 2019. Note that other high impact variants previously reported (Reuter et al., 2019) for VEGF-related genes *FLT4* (n=3) and *KDR* (n=2) do not appear here, if they were deleterious missense variants or in frame deletions, nor do variants in other VEGF genes (n=4) that were not identified/identifiable using the methods in the current study (see manuscript text for discussion), except the deletion overlapping BCAR1 (TOF220, above). Pathogenicity of variants, individual or collective, for any individual, with respect to TOF, remains uncertain.

[b] Underline indicates variants that alter evolutionarily conserved cysteine residues of *NOTCH1* (see Figure 3)

[c] Identified on reanalysis using GATK3.7 (Nov 2018).

Abbreviations: APV, absent pulmonary valve; ASD, atrial septal defect; ADHD, attention deficit hyperactivity disorder; BAV, bicuspid aortic valve; CHD, congenital heart disease; ICD, implantable cardioverter defibrillator; MAPCA, major aortopulmonary collateral arteries; PA, pulmonary atresia; PFO, patent foramen ovale; RAA, right aortic arch; SVC, superior vena cava; TOF, tetralogy of Fallot; VF, ventricular fibrillation; VSD, ventricular septal defect.
